# Population Genetic Analysis of Modern and Ancient DNA Variations Yields New Insights Into the Formation, Genetic Structure, and Phylogenetic Relationship of Northern Han Chinese

**DOI:** 10.3389/fgene.2019.01045

**Published:** 2019-10-30

**Authors:** Pengyu Chen, Jian Wu, Li Luo, Hongyan Gao, Mengge Wang, Xing Zou, Yingxiang Li, Gang Chen, Haibo Luo, Limei Yu, Yanyan Han, Fuquan Jia, Guanglin He

**Affiliations:** ^1^Center of Forensic Expertise, Affiliated Hospital of Zunyi Medical University, Zunyi, China; ^2^Department of Forensic Medicine, Zunyi Medical University, Zunyi, China; ^3^Institute of Forensic Medicine, West China School of Basic Medical Sciences & Forensic Medicine, Sichuan University, Chengdu, China; ^4^Department of Bioinformatics, WeGene, Shenzhen, China; ^5^Key Laboratory of Cell Engineering in Guizhou Province, Affiliated Hospital of Zunyi Medical University, Zunyi, China; ^6^Department of Nutrition and Food Hygiene, School of Public Health, Zunyi Medical University, Zunyi, China; ^7^Department of Forensic Medicine, Inner Mongolia Medical University, Hohhot, China

**Keywords:** ancient DNA, genetic structure, phylogenetic relationship, Han Chinese, whole-genome variations

## Abstract

Modern East Asians derived from the admixture of aborigines and incoming farmers expanding from Yellow and Yangtze River Basins. Distinct genetic differentiation and subsequent admixture between Northeast Asians and Southeast Asians subsequently evidenced by the mitochondrial DNA, Y-chromosomal variations, and autosomal SNPs. Recently, population geneticists have paid more attention to the genetic polymorphisms and background of southern-Han Chinese and southern native populations. The genetic legacy of northern-Han remains uncharacterized. Thus, we performed this comprehensive population genetic analyses of modern and ancient genetic variations aiming to yield new insight into the formation of modern Han, and the genetic ancestry and phylogenetic relationship of the northern-Han Chinese population. We first genotyped 25 forensic associated markers in 3,089 northern-Han Chinese individuals using the new-generation of the Huaxia Platinum System. And then we performed the first meta-analysis focused on the genetic affinity between Asian Neolithic∼Iron Age ancients and modern northern-Han Chinese by combining mitochondrial variations in 417 ancient individuals from 13 different archeological sites and 812 modern individuals, as well as Y-chromosomal variations in 114 ancient individuals from 12 Neolithic∼Iron Age sites and 2,810 modern subjects. We finally genotyped 643,897 genome-wide nucleotide polymorphisms (SNPs) in 20 Shanxi Han individuals and combined with 1,927 modern humans and 40 Eurasian ancient genomes to explore the genetic structure and admixture of northern-Han Chinese. We addressed genetic legacy, population structure and phylogenetic relationship of northern-Han Chinese *via* various analyses. Our population genetic results from five different reference datasets indicated that Shanxi Han shares a closer phylogenetic relationship with northern-neighbors and southern ethnically close groups than with Uyghur and Tibetan. Genome-wide variations revealed that modern northern-Han derived their ancestry from Yakut-related population (25.2%) and She-related population (74.8%). Summarily, the genetic mixing that led to the emergence of a Han Chinese ethnicity occurred at a very early period, probably in Neolithic times, and this mixing involved an ancient Tibeto-Burman population and a local pre-Sinitic population, which may have been linguistically Altaic.

## Introduction

Han Chinese, with a total population size circa 1.4 billion, is the world’s largest ethnic group and dominant ethnicity in China and Singapore. The origin of the Han Chinese population, genetic relationship with adjacent groups and past migratory pattern and admixture history have gained considerable attention from scientists working in the anthropology, linguistics, history, population and forensic genetics (Zhao et al., 2011; Gao et al., 2015; Zhao et al., 2015b; Li et al., 2017; Nothnagel et al., 2017; Zhang et al., 2017b; Chiang et al., 2018). Archaeological and anthropological evidence showed that human occupation in East Asia has experienced archaic hominin extinction, genetic introgression between early anatomically modern human and Denisovan or Neanderthals, the transformation from hunting–gathering to agriculture, massive admixture and migratory history with ethnolinguistically diverse populations in the past 50–100 thousand years (Nielsen et al., 2017). Expansions of the maternally-inherited mitochondrial DNA (mtDNA) and paternally-inherited Y-chromosome haplogroup lineages indicated that ethnically different East Asians derived from southeastern groups and experienced south-to-north migrations driven by a variety of evolutionary mechanisms (Su et al., 1999; Yao et al., 2002). Besides, social practices, including subsistence strategies, residence patterns, and agricultural expansion, play an indispensable role in shaping the patterns of Chinese populations (Nielsen et al., 2017). Ancient mitochondrial and Y-chromosomal DNA studies in East Asian Neolithic∼Iron Age populations have drastically increased in past decades (Cui et al., 2010; Li et al., 2010; Li et al., 2011; Zhao et al., 2011; Wang et al., 2012; Cui et al., 2013; Zhao et al., 2014; Dong et al., 2015; Gao et al., 2015; Li et al., 2015; Zhao et al., 2015b; Li et al., 2017; Zhang et al., 2017b; Li et al., 2018), however, how the peopling and settlement history of Neolithic populations influence the origin, expansion, and migration of the Han Chinese population is still unclear.

Physical anthropological investigation of somatometric and nonmetric features revealed that a significant difference exists between northern-Han Chinese and southern-Han Chinese (Sanchez-Mazas et al., 2011). Subsequently, Chu et al. (1998) found genetic evidence to support the distinction between southern and northern populations. Phylogeographic or genetic differentiation between northern-Han and southern-Han have been also evidenced by Yao et al. (2002) using mitochondrial DNA, Wen et al. (2004) using combined testing Y-chromosome and mitochondrial DNA variations, and Chen et al. (2009) and Xu et al. (2009) using high-density genotype data. Our previous study has investigated the genetic polymorphisms, forensic features and genetic relationship of currently widely-used autosomal short tandem repeats (STRs) in the southern-Han Chinese residing in the Pearl River Delta (He et al., 2018d). Thus, reconstructing the forensic reference database, estimating the forensic allele frequency and parameters and dissecting the genetic relationship of this genetically diverse northern-Han Chinese population are very necessary and urgent.

STR, also called as microsatellite, is one of the extraordinary mutated genetic markers, is widely existed human autosomal, X-chromosome and Y-chromosome genomes (Ge et al., 2014). This length polymorphism marker is generated by the slippage synthesis of simple sequence (2–8bp) (Schlotterer and Tautz, 1992). STRs located on the no-recombining region of Y-chromosome are the best candidates for forensic pedigree searches and identifying the perpetrator in the sexual crime or rape cases, and X-chromosomal STRs are best suitable for applications in the deficiency and incestuous cases (He et al., 2017d; Chen et al., 2018). Autosomal STR genotyping is the gold standard in the routine forensic cases. Nowadays, all organizations or countries optimized their accepted STR panels to improve the international collaboration, such as the expanded CODIS core loci, extended European standard set (ESS-extended). Huaxia Platinum System (Thermo Fisher Scientific) was integrated all twenty expanded CODIS core loci, additional STRs included in the Chinese National Database and two gender determination loci (He et al., 2018b). Single nucleotide polymorphisms (SNPs), with the number over 84.7 million in the human genome, are the best candidate to explore the detailed processes of human origin, migration, evolution, adaptation and admixture (Genomes Project et al., 2015).

Although Y-chromosomal and X-chromosomal variations of the northern-Han Chinese population have been investigated and reported (He et al., 2017d; Chen et al., 2018). Autosomal STR allele distribution of this new-generation of the Huaxia Platinum Amplification System with regard to forensic statistical features has not previously been investigated. Besides, the population genetic structure and admixture history of northern-Han *via* high-density genetic markers are unclear. Thus, we genotyped and analyzed 23 autosomal STRs in 3,089 unrelated Han Chinese individuals and 643,897 SNPs in 20 Hans residing in Shanxi Province. Shanxi Province is between 34°34′-40°44′ north latitude and 110°14′-114°33′ east longitude, which stretches about a total area of 156,700 km^2^ from the Yellow River in the west and south to the Taihang Mountain in the east and the Great Wall in the north. This area is bounded by the Shaanxi, Inner Mongolia, Hebei, Henan, and other Provinces. Archaeological, anthropological and genetic evidence from Hengbei site consistently considered that Han Chinese is originated from Shanxi and neighboring regions, also called the Central Plain (Zhao et al., 2015b). And then Han Chinese population migrated southward with the Han-associated culture (Demic diffusion) and admixed with southern Chinese natives and formed the current patterns of genetic diversity distribution (Wen et al., 2004). In addition to the estimation of forensic characterization of autosomal STRs in northern-Han, we evaluated three different population comparisons to gain a comprehensive genetic overview of the northern-Han Chinese population and nationwide and worldwide reference populations on the basis of the genetic variations of STRs (23-STRs genotype-based data set among 12 Chinese populations, 20-STRs frequency-based dataset among 53 worldwide populations and 19-STRs frequency-based dataset among 61 nationwide populations). Finally, we also collected the present available mitochondrial and Y-chromosomal genetic variations of Han Chinese populations and merged them with previously published uniparental marker variations, as well as combined whole-genome SNPs of modern and Eurasian ancient peoples, to explore the genetic legacy and phylogenetic relationship between northern Han Chinese and ancient populations (Cui et al., 2010; Li et al., 2010; Li et al., 2011; Zhao et al., 2011; Wang et al., 2012; Cui et al., 2013; Zhao et al., 2014; Dong et al., 2015; Gao et al., 2015; Li et al., 2015; Zhao et al., 2015b; Li et al., 2017; Zhang et al., 2017b; Li et al., 2018).

## Materials and Methods

### Sample Collection, Ethics Statements and DNA Preparation

This project, including study design and experimental design, was conducted in conformity with the ethical research principles for medical research involving human subjects recommended by the World Medical Association Declaration of Helsinki and approved by the Ethics Committee of Zunyi Medical University. Blood samples were collected using the FTA card with the written informed consent from 3,089 unrelated healthy Han Chinese individuals (2,009 males and 1,080 females) from Yuncheng country in the Shanxi Province, northern China. A total of 20 EDTA anti-coagulated peripheral blood samples were collected for whole-genome SNPs genotyping. All self-declared Han Chinese indigenous subjects are needed to be no intermarriage with people of other ethnic groups and resided in here at least three generations. Human genomic DNA from male samples was extracted using the DNeasy blood and tissue kit (Qiagen) and measured utilizing the NanoDrop-2000 (Thermo Scientific, USA) following the manufacturer’s instruction, and DNA from female samples was isolated using the Chelex-100 method. All our data used throughout this study are submitted in the **Supplementary Materials**.

### PCR Amplification and STR Genotyping

We employed Huaxia Platinum Amplification System for multiallelic STR genotyping including 23-autosomal-STRs, two sex-linked inherited Y-InDel (rs2032678) and Amelogenin in a ProFlex PCR System (Thermo Fisher Scientific) in accordance with the manufacturer’s recommendation. A 10 μL reaction volume, which contains 4μL of primer set (the concentration of each locus is different), 4μL of master mix, 0.4 ng of template DNA as well as Prep-N-Go buffer was employed with the standard thermal cycling conditions which comprise an initial step at 95°C for 1 min; followed by 27 cycles of denaturation at 94°C for 3 s, anneal at 59°C for 16 s, and extension at 65°C for 29 s; and following a final extension at 60°C for 5 min and preservation at 10 °C. Applied Biosystems 3500 Genetic Analyzer (Thermo Fisher Scientific) was utilized to separate and detect the PCR products using the 36 cm capillary array and POP-4 polymer following the corresponding instruction. A 9.5 μL of deionized Hi-Di formamide and a 0.5 of μL GeneScan 600 LIZ Size Standard v2.0 (Thermo Fisher Scientific) were utilized to mix with the amplified products or allelic ladder before the capillary electrophoresis. Finally, we used the GeneMapper ID-X v.1.4 software to identify and analyze the corresponding allele. We employed the typical control DNA of 9947A human cell line sample (Thermo Fisher Scientific) as positive, and the ddH_2_O as negative in each batch of PCR amplification and electrophoresis. Infinium Global Screening Array BeadChip of Illumina WeGene V2 Arrays (WeGene, Shenzhen, China) covering approximately 700k SNPs was used to genotype autosomal SNPs based on the manufacturers’ introduction.

### Datasets for Population Comparison and Statistical Analysis

To comprehensively dissect the genetic background of Shanxi northern-Han Chinese, we first integrated our raw genotyping data (23 STRs) with previously published genotype data from 11 Chinese populations from five Chinese ethnic groups (Han, Hui, Tibetan, Yi, and Uyghur) (He et al., 2018b; Wang et al., 2018a; Chen et al., 2019; Liu et al., 2019). To further explore the genetic relationship of Shanxi Han in the context of the genetic variations from the worldwide or nationwide populations, we subsequently combined our allele frequency of 20 STRs with publicly obtained data from 52 worldwide populations (Westen et al., 2012; Gaviria et al., 2013; Park et al., 2013; Fujii et al., 2014; Almeida et al., 2015; Parolin et al., 2015; Aguilar-Velazquez et al., 2016; Hossain et al., 2016; Ng et al., 2016; Park et al., 2016; Ramos-Gonzalez et al., 2016; Ristow et al., 2016; Vullo et al., 2016; Wang, 2016; Zhang et al., 2016a; Zhang et al., 2016b; Choi et al., 2017; Guerreiro et al., 2017; Jin et al., 2017; Liu et al., 2017; Moyses et al., 2017; Ossowski et al., 2017; Singh and Nandineni, 2017; Taylor et al., 2017; Wu et al., 2017; Yang et al., 2017a; He et al., 2018b; He et al., 2018e; Wang et al., 2018a; Liu et al., 2019) and allele frequency of 19 STRs with previously investigated the allele frequency distribution from 60 Chinese populations (Zhang et al., 2011; Zhang, 2012; Liu et al., 2013; Shen, 2013; Zhang, 2013; Wang, 2014; Wang et al., 2014; Xie, 2014; Hu et al., 2015; Li, 2015; Ruan, 2015; Shen et al., 2015; Wang, 2015; Yin, 2015; Zhang and Chen, 2015; Zhao et al., 2015a; Huang, 2016; Meng, 2016; Wang, 2016; Xiang, 2016; Xiao et al., 2016; Zhao, 2016; Chen et al., 2017; Fu et al., 2017; He et al., 2017a; He et al., 2017c; Jin et al., 2017; Liu, 2017; Lu et al., 2017; Yao et al., 2017; Zhang, 2017a; Zhang, 2017b; Zhang, 2017c; Zhang et al., 2017a; Zou et al., 2017; He et al., 2018b; He et al., 2018e; Wang et al., 2018a). Subsequently, to explore the genetic affinity between northern Han Chinese and ancient Asian populations, we combined mitochondrial DNA variations of 812 modern Han Chinese individuals from seven geographical different populations and 417 ancient individuals in 13 different archeological sites, and then combined Y-chromosome variations of 2,810 modern subjects from 26 Chinese populations and 114 ancient individuals in 12 neolithic sites (Cui et al., 2010; Li et al., 2010; Li et al., 2011; Zhao et al., 2011; Wang et al., 2012; Cui et al., 2013; Zhao et al., 2014; Dong et al., 2015; Gao et al., 2015; Li et al., 2015; Zhao et al., 2015b; Li et al., 2017; Zhang et al., 2017b; Li et al., 2018). Finally, we merged our 20 whole-genome SNPs data with previously published 1,924 individuals from the Human Genome Diversity Project-Centre d’Etude du Polymorphisme Humain (HGDP-CEPH) panel and International HapMap Project Phase 3 and 40 ancient humans from Eurasia (Li et al., 2008; Jeong et al., 2016; Lipson et al., 2018).

STR Analysis for Forensics (STRAF) online software (Gouy and Zieger, 2017) was utilized to evaluate the allelic frequency and forensic statistical parameters of 23 STRs. Population genetic parameters based on the raw genotype data, including pairwise Fst genetic distance, locus-specific Fst, and Fis among 12 Chinese populations, were also calculated using the STRAF. Linkage and Hardy-Weinberg equilibrium analyses, as well as evaluation of the heterozygosity indexes, were performed using the Arlequin software (version3.5) (Excoffier and Lischer, 2010). Pairwise standard genetic distances (Nei, 1978; Reynolds et al., 1983; Kalinowski, 2002) between Shanxi Han and other reference populations were calculated using the Phylogeny Inference Packages version 3.5 (PHYLIP) (Excoffier and Lischer, 2010). Principal component analysis (PCA) based on the raw data was carried out using the STRAF, and PCAs on the basis of allele frequency distribution were conducted using the Multivariate Statistical Package (MVSP) version 3.22 software (Kovach, 2007). Genetic similarities and differences revealed by the genetic distances were visualized via the heatmap using the pheatmap program in R software v3.3. Genetic relationships between Shanxi Han and other three different reference population panels were subsequently explored and reconstructed via multidimensional scaling plots (MDS) using the IBM SPSS Statistics 21 (Hansen, 2005) and neighbor-joining tree using the Molecular Evolutionary Genetics Analysis Version 7.0 (Mega 7.0) (Kumar et al., 2016). Under the ‘correlated allele frequencies’ and ‘LOCPRIOR’ models, we dissected the ancestry component among 12 Chinese populations using the STRUCTURE version 2.3.4.21(Evanno et al., 2005) with predefined populations ranging from 2∼6 with ten replications.

For genome-wide-based population genetic analyses, we used the plink v1.90 to conduct the PCA and ADMIXTURE 1.30 to perform the model-based analysis using the pruned data (-indep-pairwise 200 25 0.4). We employed 10-fold cross-validation with the predefined ancestry populations varying from 2 to 19. We conducted the *admixture-f_3_(Source1, Source2; Shanxi Han), outgroup-f_3_ (X, Shanxi Han; Yoruba), D (X, Y; Shanxi Han, Yoruba)*, *qpWave* and *qpAdm* to explore the population history of northern Han under the genetic variations of modern and ancient population using the ADMIXTOOLS (Patterson et al., 2012). We finally explored the population splits and mixtures using unsupervised clustering analysis of TreeMix (Pickrell and Pritchard, 2012).

## Results

### Genetic Structure and Population Genetic Features of Northern-Han Chinese by Raw Microsatellite Data

**Figure 1:**
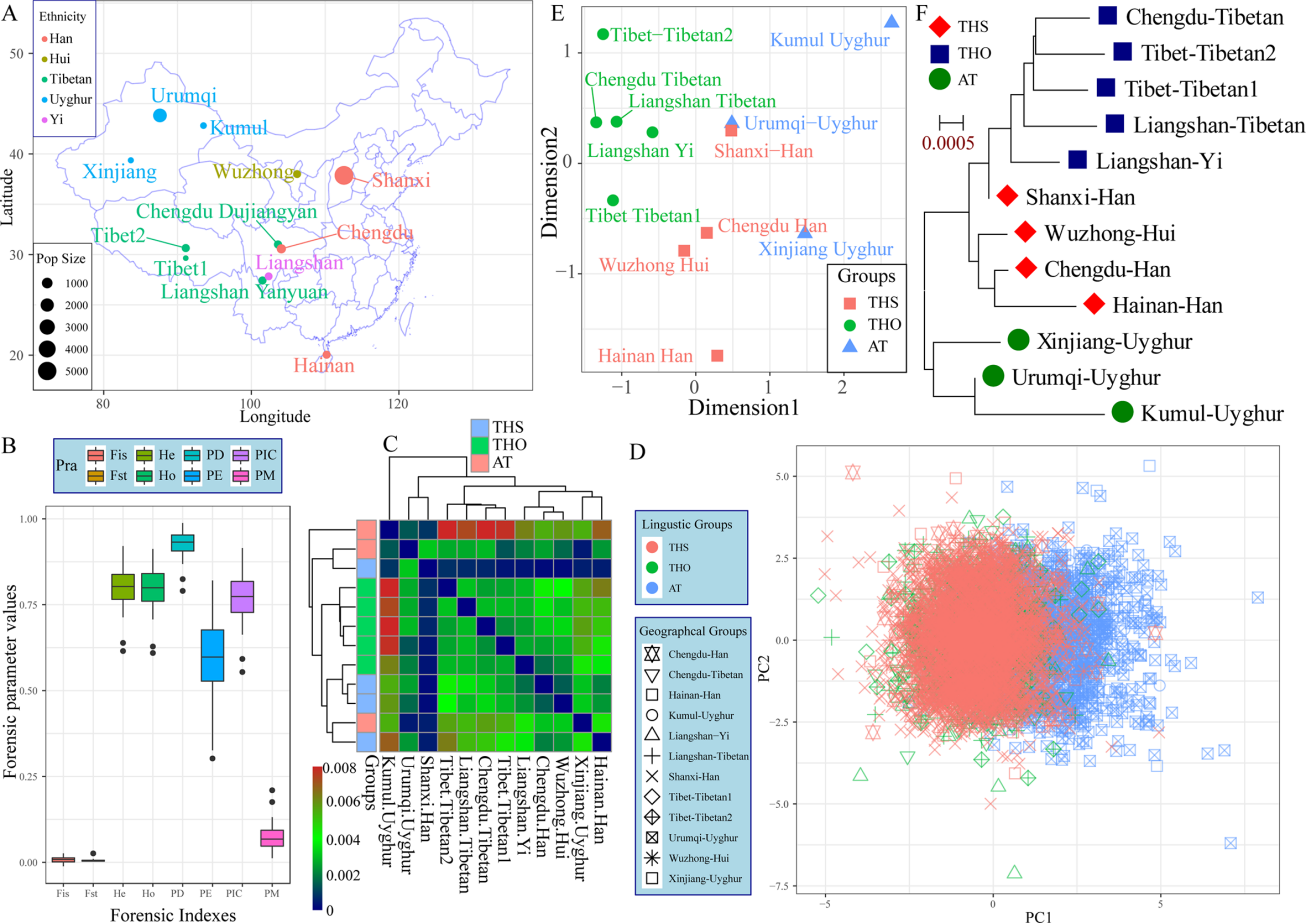
The genetic structure between Shanxi Han and other 11 Chinese populations based on the raw genotype data. **(A)** Geographic distribution, ethnicity and sample size of 12 included populations. **(B)** Forensic parameters of 23 autosomal STRs included in the Huaxia Platinum System. **(C)** Pairwise Fst genetic distance among 12 populations. PCA **(D)**, MDS **(E)** and neighbor-joining tree **(F)** show the genetic similarities and differences between newly studied Shanxi Han and other 11 reference populations. AT, Altai-Turkic; THS, Trans-Himalayan-Sinitic; THO, Trans-Himalayan except for Sinitic.

The 23 autosomal-STRs included in the Huaxia Platinum System were amplified from 3,089 northern-Han Chinese individuals residing in the Shanxi Province, which is considered as the origin place of the Han ethnic group (**Figure 1A**). The p values of Hardy-Weinberg equilibrium (HWE) and linkage disequilibrium (LD) in 23 STRs were listed in **Table S1**. After correction using the Bonferroni standard, no deviations from HWE or LD were observed. Allelic frequency distribution and forensic statistical parameters are provided in **Figure 1B** and **Table S2**. A total of 342 alleles were identified with the corresponding allele frequency ranging from 0.0002 to 0.5391. The combined power of discrimination and the combined probability of exclusion in Shanxi Han were 0.999999999999999999999999994 and 0.0.9999999995, respectively. Forensic discrimination powers estimated in the northern-Han Chinese were consistent with forensic measures in the southern-Han populations, such as Zhujiang Han and Sichuan Han. All of the combined results showed that Huaxia Platinum System was informative and polymorphic in Shanxi Han population and could be considered to be a useful tool for forensic kinship identification and individual identification, and Chinese reference database establishment.

To explore the genetic background of the Han Chinese population, we conducted the first population genetic analysis employing a genotype dataset consisting of 6,060 individuals from two language families (Trans-Himalayan and Altaic). Pairwise Fst genetic distances between Shanxi Han and other eleven Chinese populations were listed in **Table S3** and **Figure 1C**. Chengdu Han showed the smallest genetic relationship with Shanxi Han (0.0002) and Urumqi Uyghur showed the largest genetic relationship with Shanxi Han (0.0032). Pairwise Fst results showed that Shanxi Han had a closer genetic relationship with all East Asians compared to the relationships of pairs of the other Chinese populations under the same reference panel. PCA based on the first two components could explain 1.07% variance (PC1: 0.58% and PC2: 0.49%). PC1 could partly distinguish the Altaic-speaking populations and other Chinese references. Both components could not separate any ethnic group from others except for Uyghur (**Figure 1D**). MDS and neighbor-joining tree were constructed on the basis of the pairwise Fst genetic distance matrixes. As shown in **Figure 1E**, five Tibeto-Burman-speaking populations grouped together and localized in the upper left part and Sinitic-speaking populations tended to be allocated in the center. Xinjiang Uyghur and Kumul Uyghur located in the right part except for Urumqi Uygur. It is strange to find a closer genetic relationship between Shanxi Han and Urumqi Uyghur, which might be caused by the metropolitan or the provincial center with the massively genetic admixture with adjoining populations in the historical time. As we expected, three obvious genetical affinity clusters were observed which corresponding to linguistic classifications, including the Altaic-Turkic (AT) cluster, Trans-Himalayan-Sinitic (THS) cluster and other Trans-Himalayan populations (THO) cluster, and Shanxi Han was located between the THO and THS, which first grouped with the Liangshan Yi group and then grouped with Han and Hui populations (**Figure 1F**). To directly visualize the genetic component and the corresponding proportion, we conducted Structure analysis assuming 2–6 predefined ancestry populations (**Figure S1**). At k = 2, AT populations showed different ancestry components with other Chinese populations. At k = 3, THO populations showed their specific ancestry component. Geographically different ancestries gradually appeared within the same language family and shared varying proportions of each predefined ancestry when the k values continually increased. Shanxi Han consistently kept a unique ancestry component and harbored a closer genetic relationship with Chengdu Han and Wuzhong Hui than with Hainan Han.

### Genetic Heterogeneity and Phylogenetic Relationships Among Worldwide and Nationwide Populations

To characterize the general patterns of genetic similarities and differences between northern-Han Chinese and more reference populations across the whole world, we combined our newly obtained allele frequency data with previously published data from 52 worldwide populations. We calculated two pairwise genetic distances, Nei and Reynolds, among the 53 populations (**Table S4** and **Figure S2A**). Central Chinese Han showed the smallest genetic distances with Shanxi Han (Reynolds: 0.004 and Nei: 0.0013), followed by the southern-Han Chinese populations (Guangdong Han, Sichuan Han, Xiamen, and Guizhou Han), Tibeto-Burman speakers, Turkic speakers, and other continental groups in order. Heatmap furtherly illustrated the genetic affinity existing within East Asian populations (**Figure S2B**). PCA was conducted based on the allele frequency distribution of 20 STRs in 53 populations (**Figures S3** and **4**). Top ten components could extract approximately 84.40% variance (PC1: 32.126%; PC2: 15.892%; PC3: 10.083%; PC4: 8.077%; PC5: 6.523%; PC6: 3.432%; PC7: 2.55%; PC8: 2.104%; PC9: 1.857% and PC10: 1.745%). PC1 displayed a clear separation between East Asians, Central Asians, and others, and PC2 showed differences between Americans and Europeans with some exceptions. Genetic relationships were also constructed using MDS plots. The observed patterns of relationships were consistent with the aforementioned findings *via* the first three PCA components. The patterns of the genetic affinity of East Asian populations were in accordance with the linguistic classification. Southern-Han Chinese populations were well-positioned at the end of the tree and then clustered with Central-Han Chinese population, followed by clustering with northern-Han Chinese populations (Shanxi Han), finally followed by Japanese, Korean and other language family or continental populations (**Figure S3D**).

**Figure 2:**
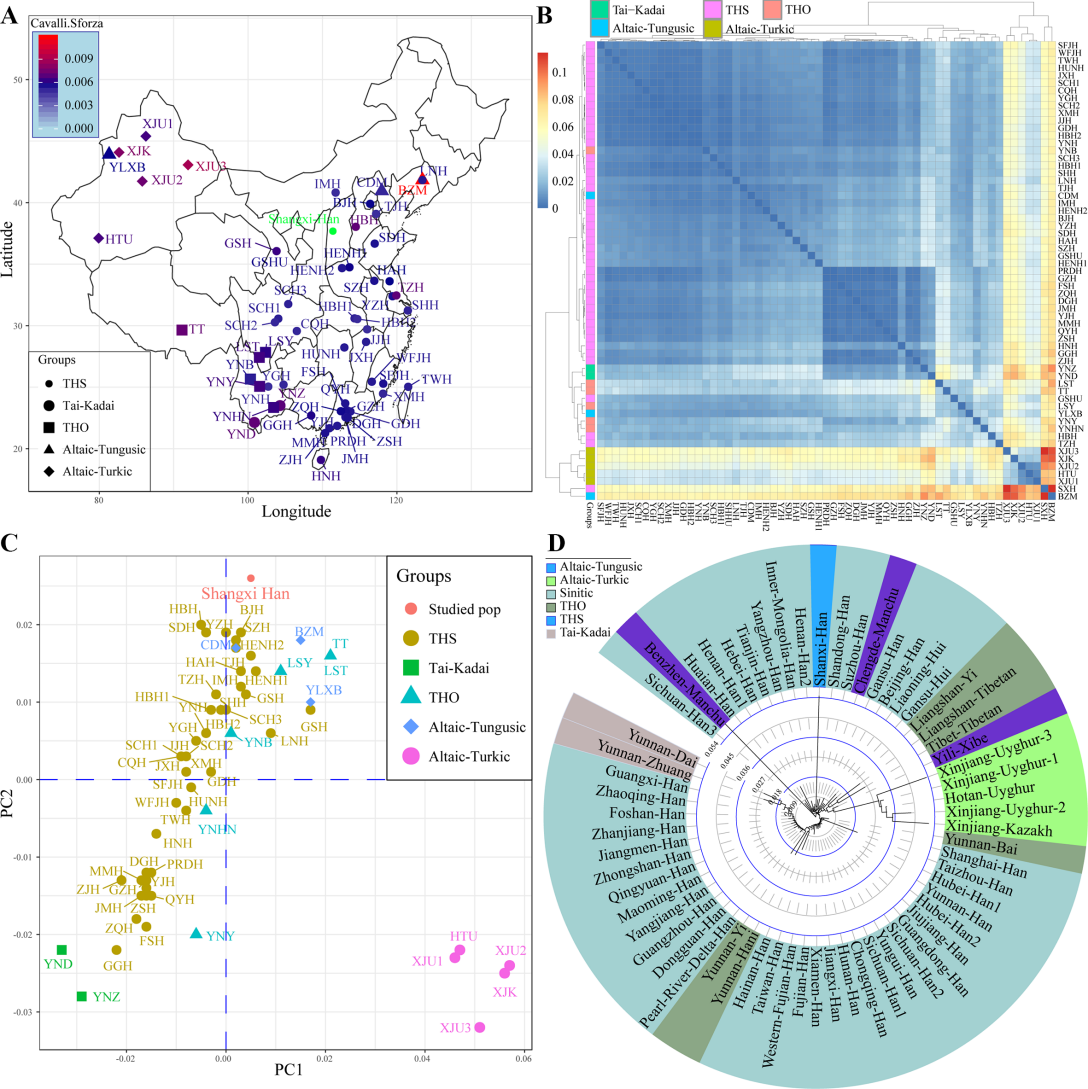
Sampling locations and pairwise Cavalli–Sforza genetic distance between Shanxi Han and other 61 Chinese reference populations **(A)**. Overview pairwise genetic distance among 62 populations from five different language families **(B)**. Principal component analysis **(C)** and neighbor-joining tree **(D)** respectively constructed on the basis of allele frequency distribution and pairwise Nei genetic distance. THS, Trans-Himalayan-Sinitic; THO, Trans-Himalayan except for Sinitic.

Subsequently, focused on the genetic variations in China, we collected previously investigated allele frequency data of 19 STRs from 61 populations and combined with our data. Results from pairwise genetic distances showed that Shanxi Han had a close genetic relationship with Tianjin Han (Cavalli–Sforza: 0.0048, Nei: 0.0514) and other geographically adjacent populations (**Figures 2A, B**). The largest genetic distances between Shanxi Han were observed with Benzhen Manchu (Cavalli–Sforza: 0.0118 and Nei: 0.1142), followed by Uyghur and Kazakh populations. MDS was constructed based on the pairwise Nei standard genetic distance matrixes (**Figure 2C**). Five AT-speaking populations were clustered together and allocated in the right-lower position and other Chinese minority groups had a scattered distribution and near to the Han Chinese populations. A significant south-to-north cline was observed and Shanxi Han was clustered closely with northern-Han populations. Phylogenetic relationships within Chinese populations were subsequently constructed (**Figure 2D**). Southern-Han Chinese and southern native minorities formed the first branch, and the AT- and THO-speaking populations formed the second branch. Northern-Han Chinese and northern Manchu groups formed the third branch. Shanxi Han was localized between the neighboring northern populations, such as Yanzhou, Inner Mongolia, Henan2, Shandong and Suzhou Han Chinese populations. The results from different standard genetic distances, PCA, MDS, and phylogenetic relationship reconstruction consistently indicated that Shanxi Han carried a higher resemblance to Beijing Han Chinese than with southern-Han Chinese or other THO-, AT-, Hmong-Mien- and Tai-Kadai-speaking populations.

### Haplogroup-Based Meta-Analysis of Ancient and Modern Human Populations

**Figure 3:**
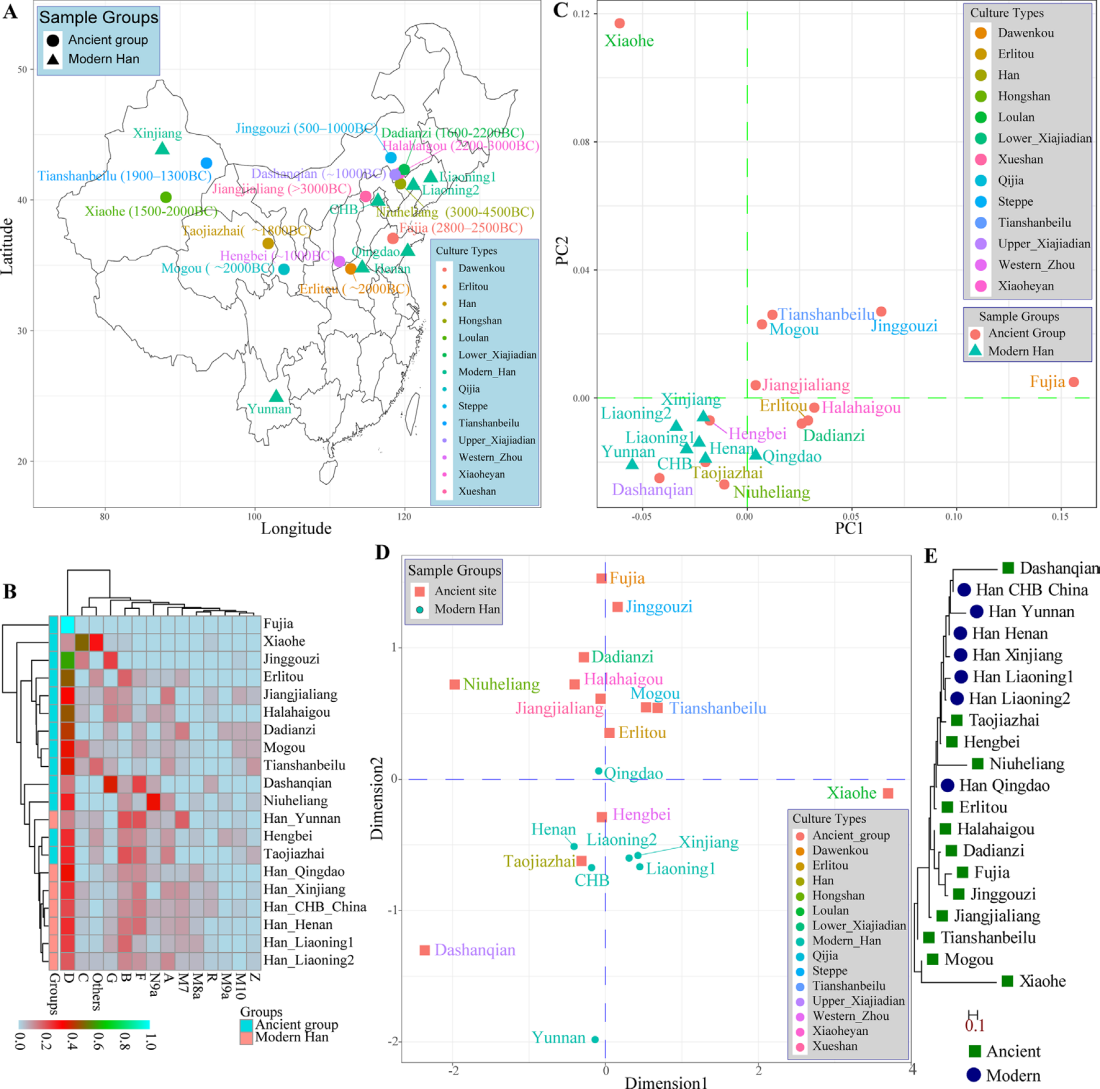
The genetic affinity between ancient Chinese populations and modern Han Chinese populations based on the mitochondrial variations. **(A)** Geographical positions, culture types and calibrated years of 13 ancient archeological sites and geographical information of seven modern Han Chinese populations. **(B)** Heatmap of pairwise Nei genetic distance between ancient and modern populations. **(C)** Principal component analysis among 20 populations. **(D)** Two-dimensional scaling plot results reconstructed based on the Nei genetic distances. **(E)** Neighbor-joining tree of Nei distances calculated based on mitochondrial genetic variation.

A large number of archeological and historical evidence supported that modern Han Chinese population was derived from the central plain area, near to Shanxi Province, and subsequently admixed with neighboring minority groups (Zhao et al., 2014; Zhao et al., 2015b). Fortunately, many genetic studies focused on the genetic architecture of Chinese ancient populations from Neolithic, Bronze, and Iron Age using the uniparental genetic markers (MtDNA and Y-chromosome) had been performed. Therefore, we comprehensively assessed the genetic difference and phylogenetic relationship between modern and ancient Chinese populations using the publicly available prehistoric Chinese archeological samples. We first meta-analyzed mitochondrial variations using 417 ancient samples assembling from 13 archeological sites (Jiangjialiang, Niuheliang, Halahaigou, Dadianzi, Dashanqian, Erlitou, Hengbei, Taojiazhai, Mogou, Xiaohe, Tianshanbeilu, Fuji, and Jinggouzi) and 770 modern northern-Hans from Beijing Han in the 1,000 genomes project and other genetic studies (Henan, Liaoning, Qingdao, and Xinjiang) and 42 southern-Han samples from Yunnan Province. Geographical position and corresponding calibrated ages of archeological sites were presented in **Figure 3A**. We assessed the haplogroup frequency distribution and measured the genetic distances between northern-Han Chinese and adjacent ancient populations. Significant mitochondrial haplogroup frequency differences between modern and ancient groups except for Hengbei and Taojiazhai populations were observed (**Figure 3B**). The relatively smaller genetic distances between ancient and modern populations were observed between Erlitou, Hengbei, Taojiazhai, and other modern Han Chinese populations. The top two components revealed 69.322% variance from 25 populations. Xiaohe population was isolated from other modern and ancient populations, which was consistent with their origin from admixture between European and East Asian (**Figure 3C**) (Li et al., 2015). Hengbei, Taojiazhai, Niuheliang, and Dashanqian populations had a close relationship with modern Han populations. Similar patterns of population distribution model were also reported in the MDS results (**Figure 3D**). Southern Han Chinese from Yunnan kept a separate relationship from the northern-Han Chinese. The phylogenetic relationship between modern and ancient populations in **Figure 3E** showed a close relationship between Dashanqian ancient population and Beijing Han Chinese and a close relationship between aforementioned three archeological sites (Hengbei, Taojiazhai, and Niuheliang). The Erlitou ancient population was close to Qingdao Han. These ancient populations might be the ancestry population of modern Han Chinese.

To further evaluate the genetic ancestry and genetic relationship between northern-Han and ancient populations from paternal inherited legacy, we collected data from 114 samples obtained from 12 ancient archeological sites (Jiangjialiang, Sanguan, Niuheliang, Halahaigou, Dadianzi, Dashanqian, Jinggouzi, Miaozigou, Hengbei, Taojiazhai, Mogou, and Tianshanbeilu) and then combined with modern data from 2,810 individuals. A total of 80.6% variance extracted from 38 populations showed population substructure existed among the modern Han Chinese population. And Hengbei, Jinggouzi, Dashanqian, Mogou, Taojiazhai, and Sanguan ancient populations showed a paternally close relationship with the modern Han Chinese populations (**Figure S5A**). Fst and corresponding p values between different populations were calculated and submitted in **Table S6**. Significant Y-chromosome haplogroup frequency differences were observed due to the statistically significant differences among 572 out of 703 population comparison pairs. MDS was constructed on the basis of the linearized Fst genetic distance matrix (**Figure S5B**). Nine ancient populations were localized in the upper right position and the other three groups were allocated in the left-center part. Modern Han Chinese populations were clustered in the center and right lower positions. We reconstructed phylogenetic neighbor-joining tree on the basis of the pairwise linearized standard distance, which showed two different clusters: one mainly consisted of modern Han Chinese populations and the other comprised three Taiwan populations, one China Han Chinese, and all ancient populations with the exception of the Hengbei ancient population (**Figure S5C**). The Hengbei ancient population was first clustered with Shanxi and other northern Han Chinese populations (Shaanxi and Heilongjiang).

### High-Density Genetic Variations of Modern and Ancient Genomes Show Fine-Scale Population Structure of Northern Han

**Figure 4:**
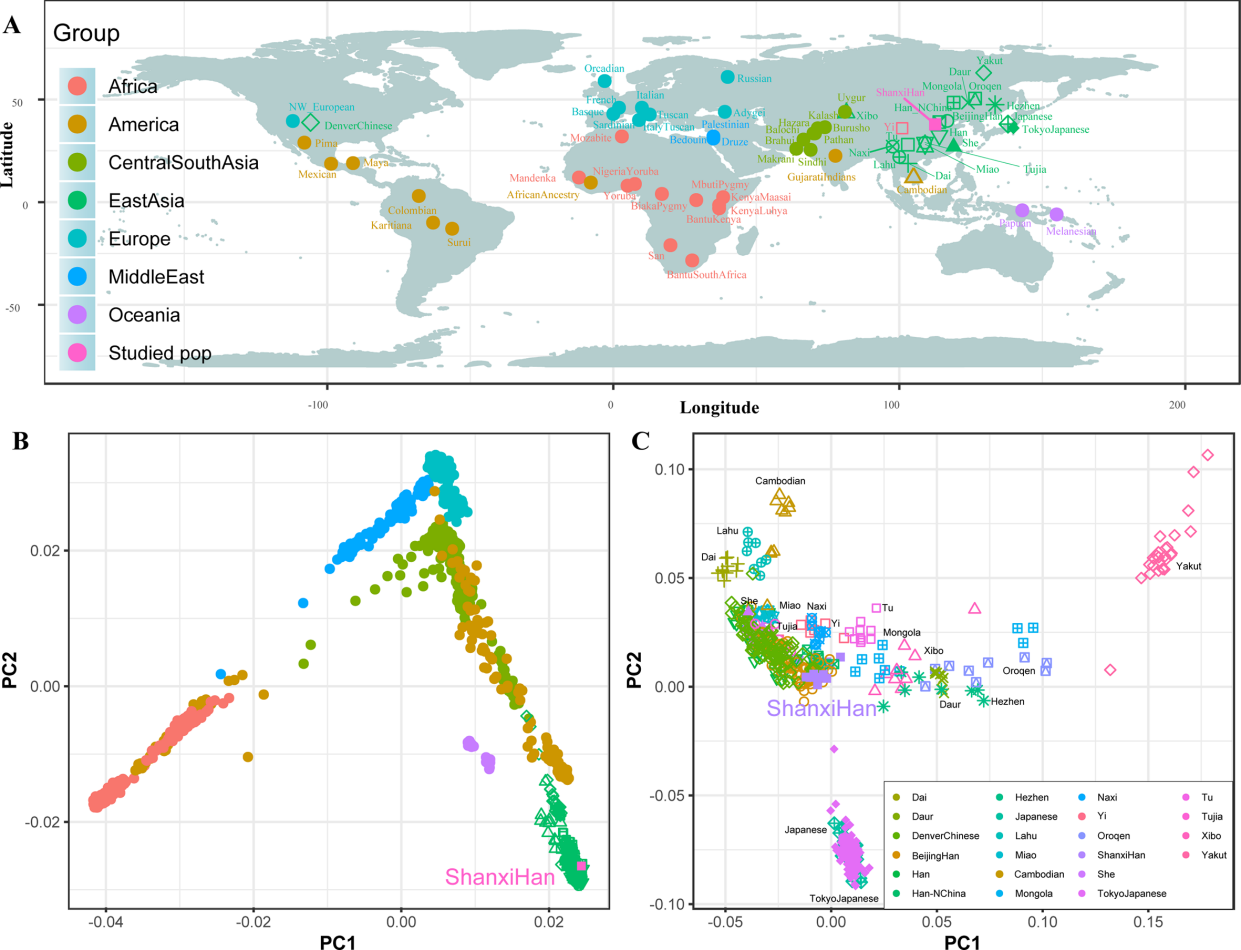
Geographic position **(A)** and PCA results **(B** and **C)** among 65 worldwide populations.

**Figure 5:**
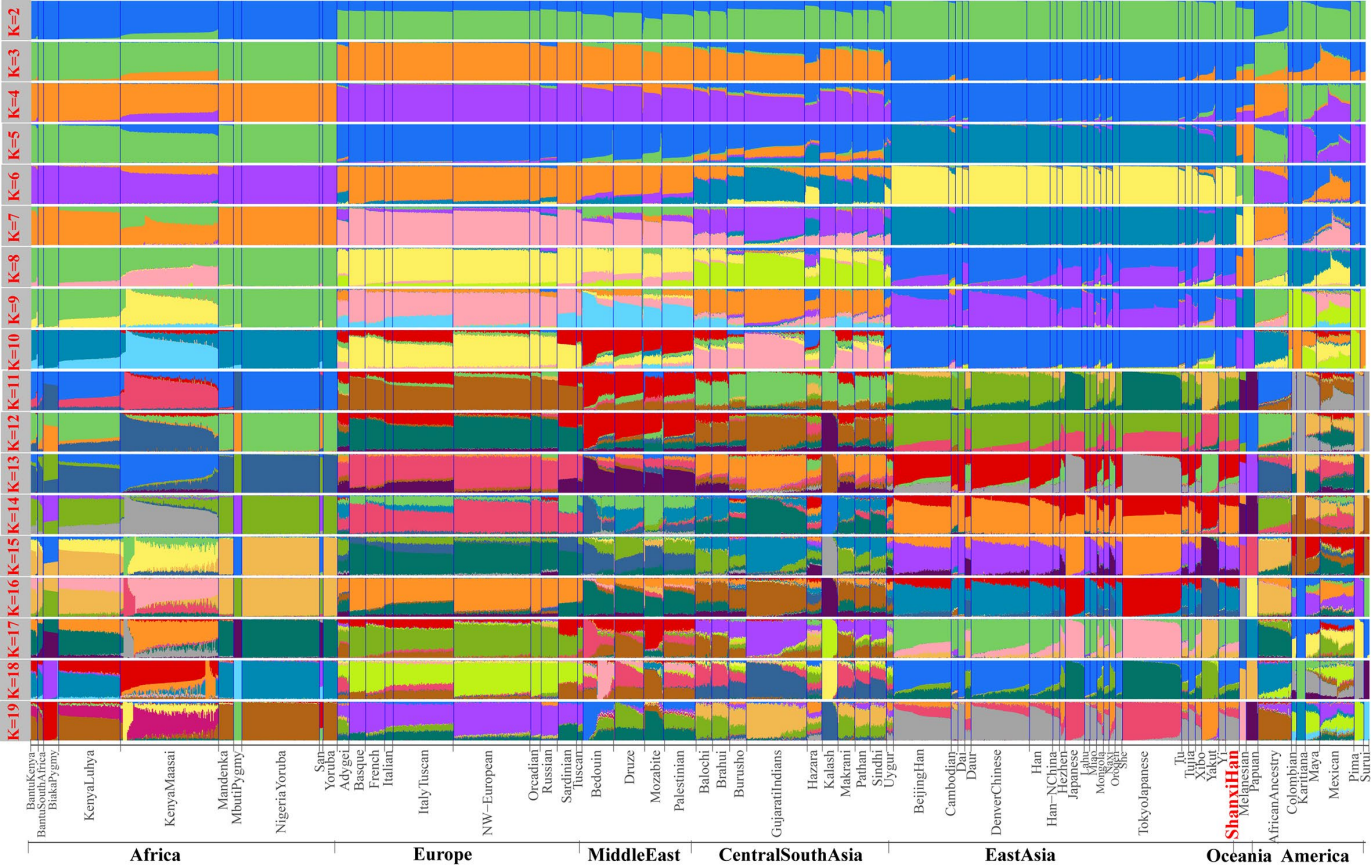
Model-based results of 65 populations with predefined ancestry populations varying from 2 to 19.

**Figure 6:**
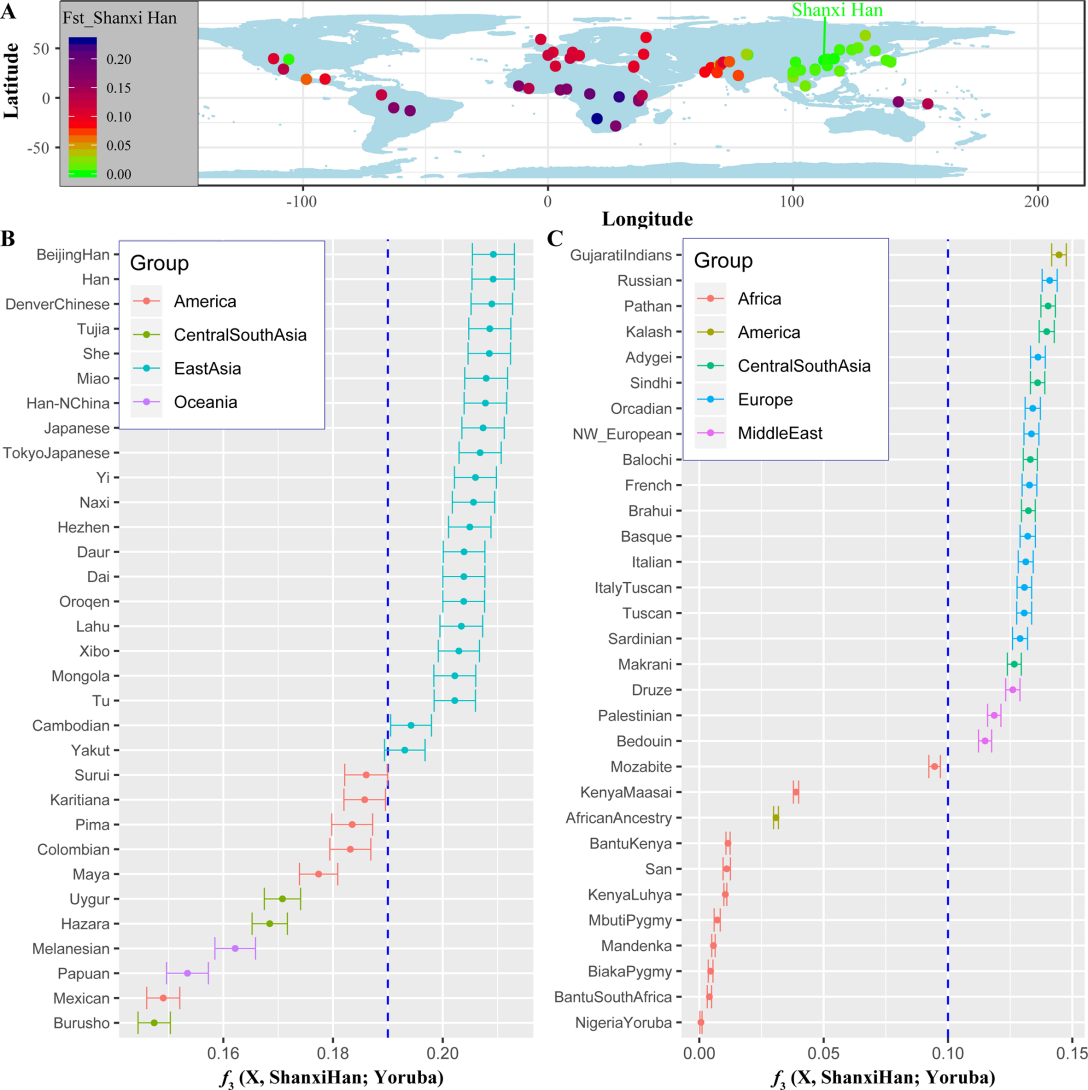
The genetic affinity between Shanxi Han and other 64 worldwide populations revealed by pairwise Fst genetic distance **(A)**, shared alleles **(B** and **C)**.

**Figure 7:**
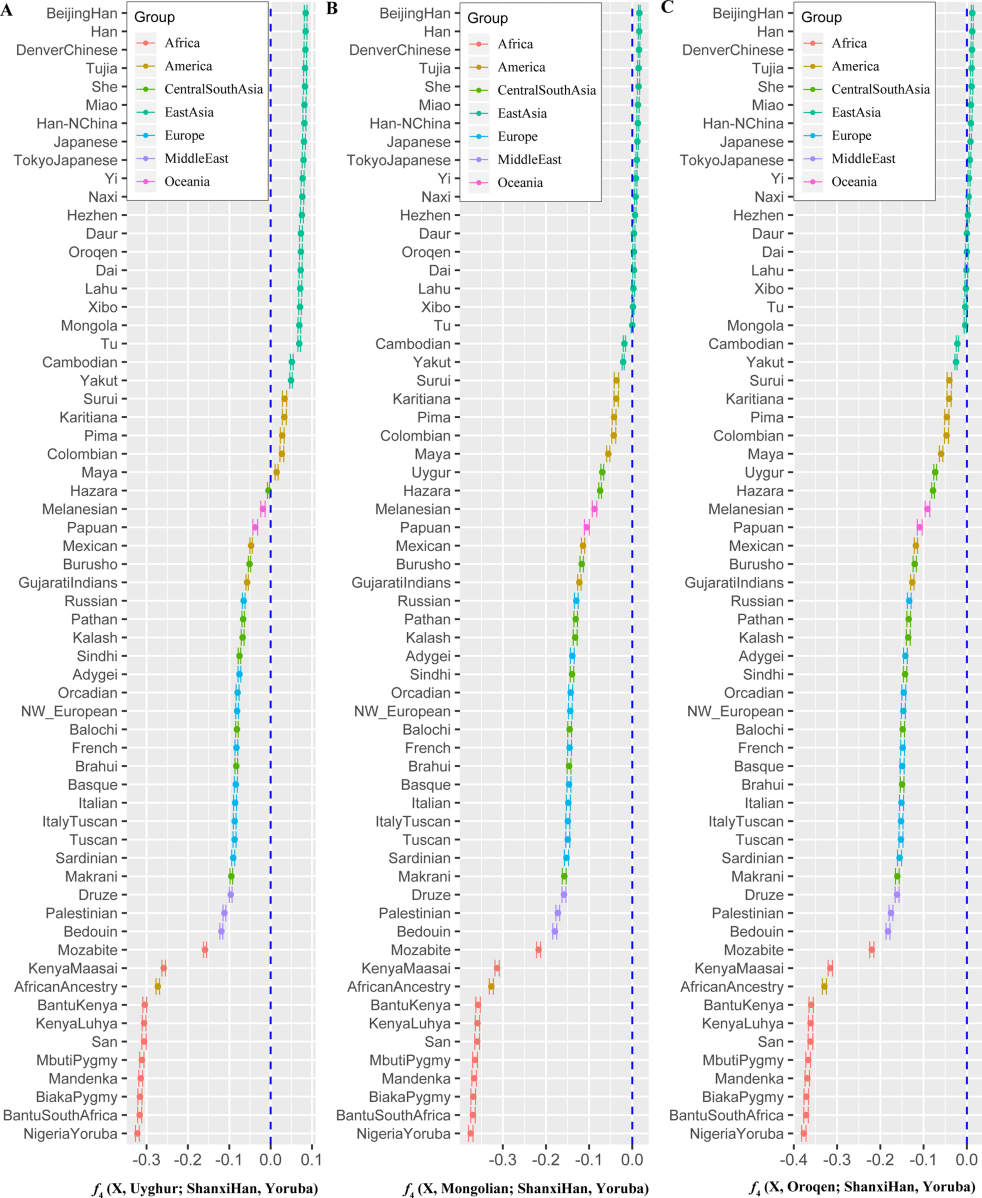
Shared genetic components with Shanxi Han between Altai-speaking populations and other worldwide reference populations: Turkic-speaking Uyghur **(A)**, Mongolic-speaking Mongolian **(B)** and Tungusic-speaking Oroqen **(C)**.

**Figure 8:**
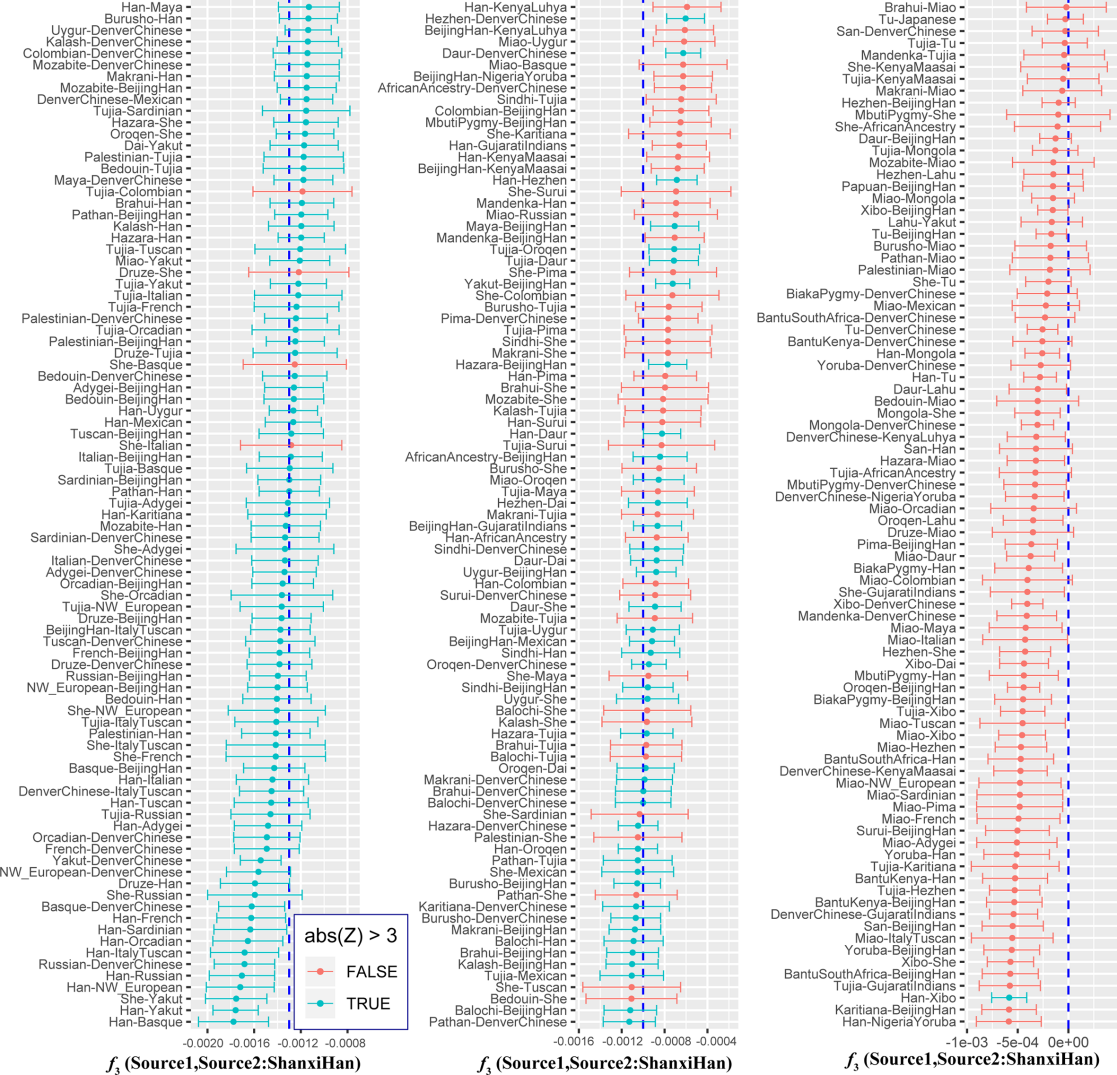
Admixture *f*_3_ results with significant negative *f*_3_ values.

We additionally investigated the fine-scale genetic structure of Shanxi Han by determining the genetic relationships under the context of 65 worldwide populations (**Figure 4A**). PCA of worldwide populations allocated Shanxi Han at the end of the Eurasian–American genetic cline (**Figure 4B**). And Shanxi Han clustered closely with Beijing Han in the finer scale of variations from East Asia (**Figure 4C**). The observed patterns of genetic affinity were subsequently supported by the results from ADMIXTURE analysis (**Figure 5**). East Asians were a homogeneous population when the predefined ancestry populations are less than eight. Genetic component kept similar between Shanxi Han and Beijing Han, Denver Chinese, Han, and northern Han. The pairwise Fst genetic distances estimated using SNP data were presented in **Table S7**. The smallest genetic distance was identified between Shanxi Han and Beijing Han (Fst = 0.0006), followed by Tujia and another northern-Han (**Figure 6A**). *Outgroup-f_3_* in the form of *f_3_ (X, Shanxi Han; Yoruba)* showed that the greater genetic affinity identified between Shanxi Han and Han Chinese populations, subsequently followed by the southern Tai-Kadai and Hmong-Mien speakers, and Western Trans-Himalayan and northern Altaic speakers (**Figures 6B, C**). We subsequently estimated the *D-statistics* in the form of *D (X, Y; Shanxi Han, Yoruba)*, where X represents the worldwide populations and Y denotes the Chinese populations from different language families, to explore the status of allele sharing. Our results provided supporting evidence for more shared genetic drift between Shanxi Han and northern-Han or neighboring minorities (**Figure 7** and **S6** and **S7**). We used the admixture *f*_3_*(Source1, Source2; Shanxi Han)* to find the potentially admixed ancestral populations. Two hundred sixty-four out of 2,016 pairs were observed with significant negative values (**Figure 8**). The potential ancestry populations revealed by the *admixture-f_3_* indicated that the ancestral populations of Shanxi-Han derived their ancestry from southern Chinese-related population (Ancestral Southeast Asians) and East-Siberian-related population (Ancestral Northeast Asians), like the two ancestry populations observed in Indian (Ancestral North Indians and Ancestral South Indians) (Reich et al., 2009).

**Figure 9:**
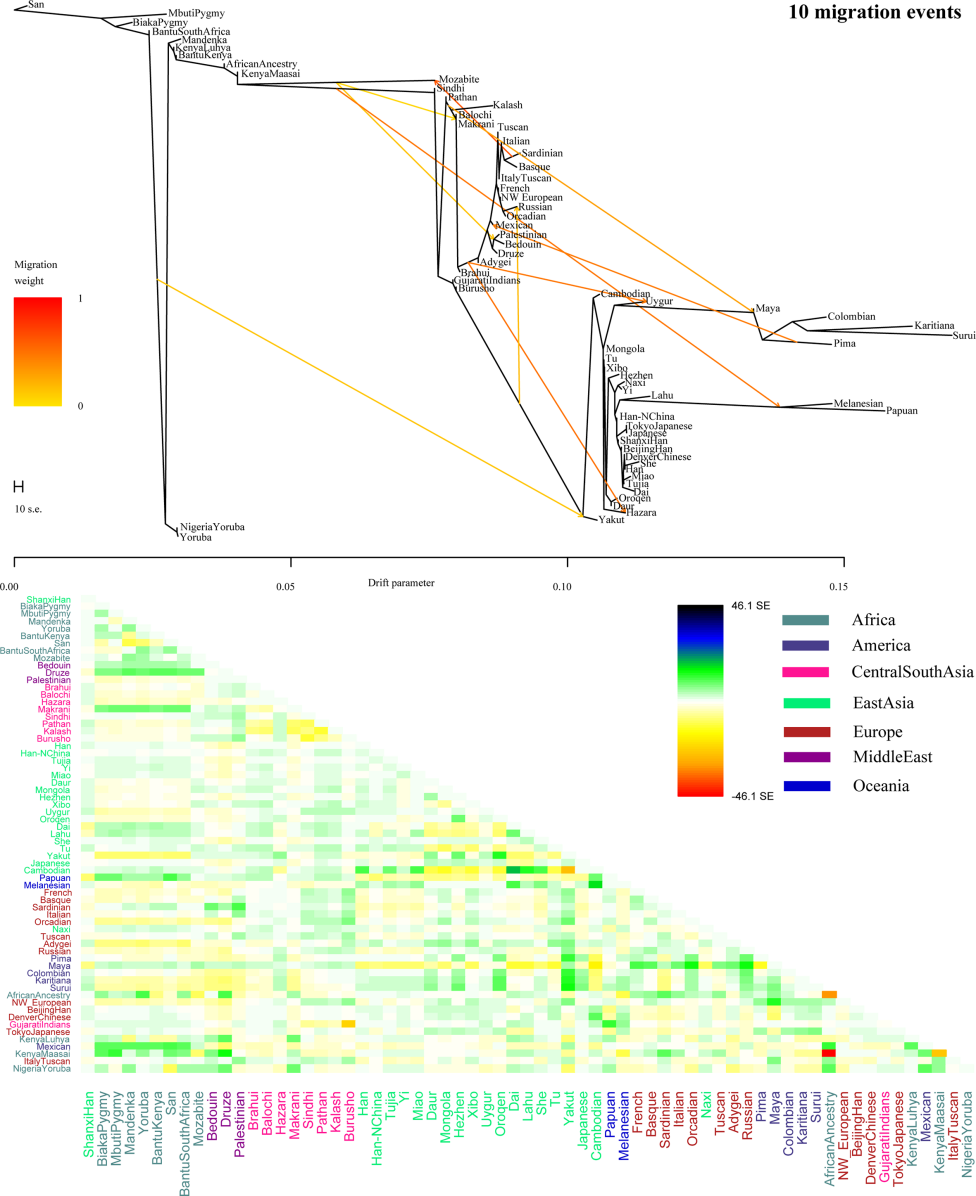
Population splits and admixtures among 65 populations with a prior assumption with 10 migration events inferred from ML tree and model residual. The top panel represents the ML tree with the ten migrations and the bottom panel shows corresponding model residuals.

To further validate the minimum streams of ancestry populations and evaluate the corresponding admixture proportion, we first performed the TreeMix analyses among 65 worldwide populations (**Figures 9** and **S8**) and 25 Asian populations (**Figure S9**). A larger number of recent admixtures or migrations were observed in our TreeMix model. Considering the statistical significance (*f_3_* = −0.0018 and Z = −6.662) was observed in the form of *f*_3_*(She, Yakut; Shanxi Han)*, we then conducted the *qpWave* and *qpAdm* using the She and Yakut as source populations and using Yoruba, San, Papuan and Melanesian as outgroup populations. *QpWave* results (p = 0.052 for rank1) indicated that Shanxi Han was derived from two ancestral populations. The *qpAdm* analysis further suggested that Shanxi Han has derived 25.2% Yakut-related ancestry and 74.8% She-related ancestry.

**Figure 10:**
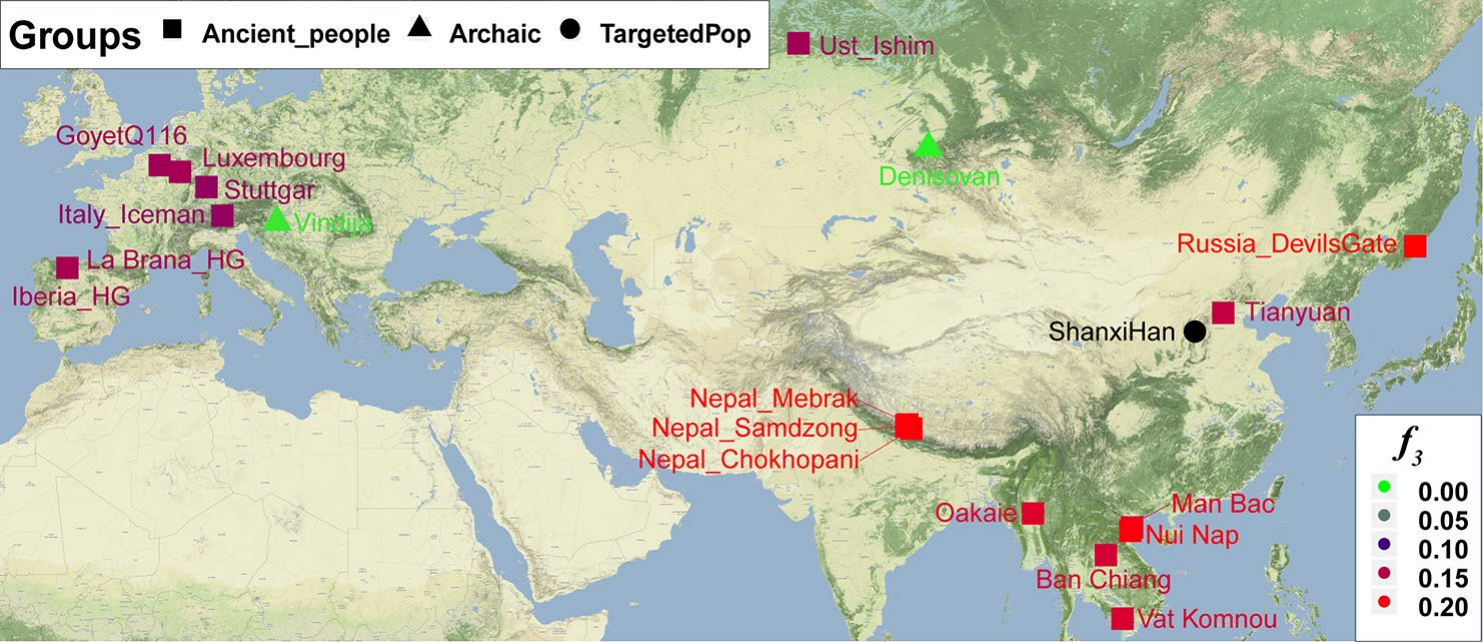
The genetic affinity between Shanxi Han and 19 ancient populations revealed from *f*_3_-statistics.

Nineteen ancient populations from Eurasian were employed to explore the genetic admixture history between Shanxi Han. The shared genetic history of all pairs was presented in **Figure S10**. Denisovan and Vindija Neanderthal shared smaller genetic components with others. We found that Shanxi Han kept a distant genetic relationship with two archaic human populations and Italy Iceman, and shared more alleles with DevilsGate (0.2039) and other ancient populations from Southeast Asia and Nepal (**Figure 10**). DevilsGate, Oakaie showed significantly negative *f*_3_ value in the form of *f*_3_*(A, B; Shanxi Han)* (**Table S8**). We finally used *qpWave* to find that the minimum ancient ancestry streams modern northern-Han were 2 (rank1: p = 0.118). *QpAdm* further showed that DevilsGate Hunter-Gatherer-related population contributed 45% ancestry and Oakaie-related ancient population contributed 55% ancestry to modern northern-Han Chinese.

## Discussion

East Asia is occupied by anatomically modern human 50 kya when they migrated out of Africa. These regions are populated by the hunter–gatherer over 40 kya in the Paleolithic time (Nielsen et al., 2017). Under the natural selections from different environments, substance strategies and disease pathogens, the different ethnic group formed their specific genetic structure with a different culture, appearance, and language. In the Neolithic time, agriculture originated from the Yellow, Yangtze and Zhujiang River Basins, may be also included in Liaohe, promoted the process of population genetic structure change with different cultures (Piao et al., 2010). For China in East Asia, the world’s largest ethnic group of the Han Chinese, 55 officially recognized and several unrecognized ethnic groups are subsequently formed with their specific cultural background. The languages they used in this region include over nine language families: Indo-European, Altaic (also called “Trans-Eurasian,” including Turkic, Mongolic, and Tungusic language groups) mainly distrusted in the north; Tai-Kadai, Hmong-Mien, Austronesian, Austroasiatic, and Trans-Himalayan language families in the south. Population substructures among Chinese modern populations revealed by our autosomal STR panel have supported the patterns of population relationship found by the X and Y-chromosome markers, as well as ancestry-informative single nucleotide polymorphisms (He et al., 2017b; He et al., 2018a; He et al., 2018c). Here, our study presented the first comprehensive genetic analysis, including autosomal STR, a meta-analysis of mitochondrial and Y-chromosomal haplogroup distribution, and autosomal SNP data of northern-Han Chinese residing in Shanxi Province. We performed the comprehensive population comparison to investigate the origin, genetic legacy and detailed genetic relationship of modern Han Chinese population, especially for the northern Han Chinese. Our results showed that Altaic and Trans-Himalayan speakers except for Sinitic speakers harbored considerable genetic differences with Han Chinese populations. However, no apparent genetic differentiation between Hmong-Mien-, Tai-Kadai and neighboring Han Chinese populations is revealed in our present study (**Figure 1**). Analysis from the haplogroup distribution of Neolithic Chinese populations showed that the significant association of genetic continuity between ancient populations from Yellow River Valley sites (Mogou, Taojiazhai, and Hengbei) and modern northern-Han Chinese (**Figures 2** and **S5**). Whole-genome high-density SNP data illustrate that Shanxi Han Chinese inherited 25.2% their ancestry from Yakut-related population and 74.8% from She-related population. Ancient autosomal genetic variation subsequently shows a two-way admixture from ancient North East Asian (45% ancestry from DevilsGate Hunter-Gatherer-related population) and ancient South Asian (55% ancestry Oakaie-related ancient population). These results consistently showed a more complex and ancient population admixture history of northern Han Chinese. Han Chinese may be originated from the admixture between the ancient Tibeto-Burman population and a local pre-Sinitic population which may have been linguistically Altaic in the Neolithic time when agriculture emerged in Yangtze and Yellow River Basins.

A recent large-scale whole-genome variation study covering 11,670 Han Chinese individuals from 24 out of 33 administrative regions was carried out to explore the Han Chinese population genetic structure and genetic ancestry (Chiang et al., 2018). Their valuable finding of east-west genetic distinction among Han Chinese is one indispensable previously unrecognized population structure, which is perfectly complemented the north-south differentiation previously found by Xu et al. (2009). Using high-density SNP typing data and other scientists using uniparental markers and classical markers (Xu et al., 2009; Stoneking and Delfin, 2010; Sanchez-Mazas et al., 2011). Our results in this study on the basis of the nationwide STR variations also provide the microsatellite evidence for north-south genetic cline but fail to reveal the east-to-west difference, which may be caused by the sample coverage. Lu et al. (2016) whole-genome sequenced 39 Han Chinese and 38 Tibetan individuals to investigate the gene pool of the Tibetan and Han group. They found that Tibetan and Han Chinese diverged from each other at 7,000–13,000BC during the last glacial maximum (Lu et al., 2016). Wang et al. (2018) recently also tried to investigate and elucidate the precise divergence time, genetic structure and admixture history between Han Chinese and neighboring country populations (Japanese and Korean). Their results suggested that Han Chinese and other focused two populations split approximately 1,000–1,600 BC in the Shang dynasty in Chinese history and subsequently substantial genetic admixture between them and other adjacent populations have occurred (Wang et al., 2018b).

The processes of ancient whole-genome DNA studies with the technological innovations of DNA hybridization enrichment and next-generation sequencing has revolutionized the phylogenetic relationship and population history reconstruction in the European, American, Oceanian and even southeast Asians (Nielsen et al., 2017). In East Asia, just two projects respectively focused on one 40,000-year-old individual from Tianyuan cave and two hunter–gatherers from Devil’s Gate have been performed. Yang et al. (2017) sequenced the whole-genome of Tianyuan ancient people (40,000BP) and discovered a strong genetic affinity between these ancient people and present populations, which indicated that there is a genetic continuity or population turnover in the East Asian continent (Yang et al., 2017b). Siska et al. (2017) genome-wide analyzed two Devil’s Neolithic individuals (∼7,700BP) near to the Amur basin and also detected the genetic continuity in northeast Asia. If ancient people from Paleolithic, Neolithic, Bronze, and Iron Ages in East Asia are all sequenced and conducted corresponding population history reconstruction combined with the historical, cultural, linguistic and archeological findings, a complete genetic landscape of the East Asians will be obtained. However, a number of ancient people excavated from different archeological sites in China have so far received little attention. Fortunately, there still some exploratory projects focused on the genetic variations of the uniparental markers (mtDNA and Y-chromosome) and Neolithic or historical ancient people been carried out. Thus, we can perform the first meta-analysis to investigate the phylogenetic relationship between the ancient population and modern northern-Han Chinese population.

Our present meta-analysis results from the Neolithic ancient people and modern Han Chinese on the basis of the combined genetic variations of mtDNA and Y-chromosome first showed that the ancient populations from West Liao River Valley sites (Dasanqian and Niuheliang) and Yellow River Valley sites (Hengbei, Taojiazhai) share considerable similar mitochondrial haplogroup with the modern northern-Han Chinese populations. For Y-chromosome variations, ancient people from the Hengbei site shared the more significant genetic similarity with modern northern-Han Chinese from Shanxi and Heilongjiang provinces, and Dashanqian and Mogou ancient people bear a similar genetic assemblage with modern Taiwan Han people. Mogou site in the Ganqing region adjacent to the central plain is the hometown of Di-Qiang people who are thought as the direct ancestral population of Han Chinese, which is genetically close to the Han Chinese population. Our results reveal a close genetic relationship among Hengbei, Mogou and modern northern-Han Chinese populations. Our findings combined with the archeological, historical and linguistic evidence consistently supported the admixed genetic origin of modern Han Chinese.

## Perspective

In summary, we genotyped 23-autosomal-STRs in 3,089 Shanxi northern-Han Chinese individuals and provided the first batch of allele frequency, forensic and population genetic parameters of northern Han Chinese. Comprehensive worldwide and nationwide population comparisons not only showed that Shanxi harbored a strong similar genetic assemblage with adjacent Han populations but also illustrated that there were apparent genetic distinctions between north-to-south Han Chinese as well as genetic differentiation between populations belonging to different language families, obviously differences observed between Tibetan, Uyghur, and others here. The first meta-analysis based on the mitochondrial and Y-chromosomal genetic variations among ancient and modern Asian populations showed a genetic affinity and genetic continuity between Mogou, Hengbei ancient population and present-day northern-Han Chinese. We also found Neolithic agriculture expansion related Dashanqian and Niuheliang ancient populations are genetically close to modern northern Han. The qpWave/qpAdm modeling further revealed that modern northern Han Chinese carried 74.8% She-related ancestry and 25.2% Yakut-related ancestry. Both Hengbei-associated and Tibetan-related uniparental lineage (D haplogroup) were observed in modern Northern Han Chinese. Besides, approximately 45% DevilsGate-like ancestry, one Tungusic-affiliated Neolithic population, was modeled *via* ancient DNA. Summarily, consistent with previous linguistic and archaeological evidence, the genetic mixing that led to the emergence of a Han Chinese ethnicity occurred at a very early period, probably in Neolithic times, and this mixing involved an ancient Tibeto-Burman population and a local pre-Sinitic population, which may have been linguistically Altaic. Fine-scale population history reconstruction of north Han from modern and ancient genomes consistently model their ancestral populations deriving from ancestral North East Asian and ancestral South East Asian.

## Ethics Statement

This study was approved by the Ethics Committee of Zunyi Medical University and corresponding experiments have followed the recommendations of the World Medical Association Declaration of Helsinki. Informed consent was obtained before the sample collection from the participants.

## Author Cntributions

GH, PC, and FJ conceived the idea for the study. GH, PC, and FJ performed or supervised wet laboratory work. PC, JW, LL, HG, XZ, HL, LY, GH, MW, YH, GC, and YL analyzed the data. GH wrote and edited the manuscript.

## Funding

This work was supported by grants from the PhD Scientific Research Start-up Fund of Affiliated Hospital of Zunyi Medical University (No. 201501) and the National Natural Science Foundation of China (No. 81401562).

## Conflict of Interest

The authors declare that the research was conducted in the absence of any commercial or financial relationships that could be construed as a potential conflict of interest.
