# Supplementary material for: Population Genetic Analysis of Modern and Ancient DNA Variations Yields New Insights Into the Formation, Genetic Structure, and Phylogenetic Relationship of Northern Han Chinese
Source: Front Genet. 2019 Oct 30;10:1045. doi: 10.3389/fgene.2019.01045 (PMC6832103; doi:10.3389/fgene.2019.01045)
Supplement: Supplementary file 1 [file DataSheet_1.pdf]

Supplementary Figures S1~10

Population Genetic Analysis of Modern and Ancient DNA Variations Yields New Insights into the Formation, Genetic Structure and Phylogenetic Relationship of Northern Han Chinese

Pengyu Chen[1,3,+], Jian Wu[1,3,+], Li Luo[1,3,+], Hongyan Gao[1,3], Mengge Wang[2], Xing Zou[2], Yingxiang Li[4], Gang Chen[4], Haibo Luo[3], Limei Yu[5], Yanyan Han[6], Fuquan Jia[7,*], Guanglin He[2,*]

[1]Center of Forensic Expertise, Affiliated Hospital of Zunyi Medical University, Zunyi, Guizhou, China
[2]Institute of Forensic Medicine, West China School of Basic Medical Sciences & Forensic Medicine, Sichuan University, Chengdu, Sichuan, China
[3]Department of Forensic Medicine, Zunyi Medical University, Zunyi, Guizhou, China
[4]Department of Bioinformatics, WeGene, Shenzhen, China
[5]Key Laboratory of Cell Engineering in Guizhou Province, Zunyi, Affiliated Hospital of Zunyi Medical University, Guizhou, China
[6]Department of Nutrition and Food Hygiene, School of Public Health, Zunyi Medical University, Zunyi, Guizhou, China
[7]Department of Forensic Medicine, Inner Mongolia Medical University, Hohhot, Inner Mongolia Autonomous Region, China

+ These authors contributed equally to this work.

* Correspondence and requests for materials should be addressed to
Fuquan Jia (jiafuquan915@163.com)
Guanglin He (Guanglinhescu@163.com)

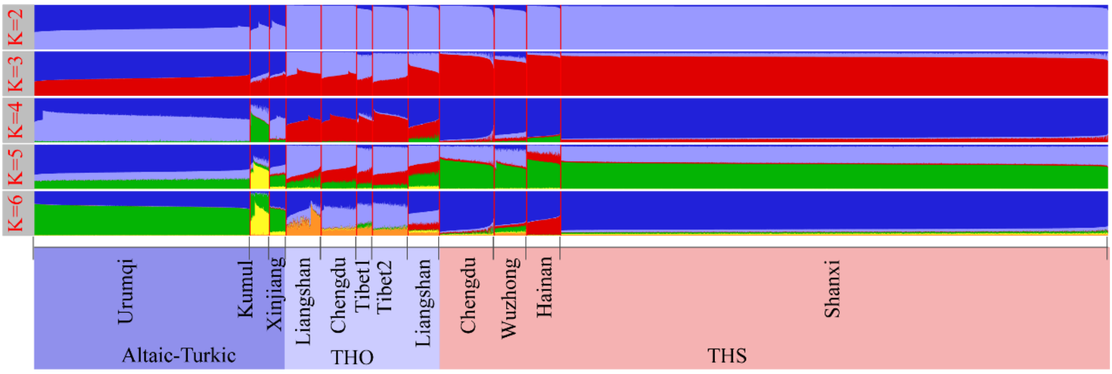

Figure S1. Structure results of 12 Chinese populations projected on the genetic variations of 23 autosomal STRs in 12 Chinese populations belonging to Turkic, Tibeto-Burman and Sinitic language family. THS: Trans-Himalayan-Sinitic; THO: Trans-Himalayan except for Sinitic.

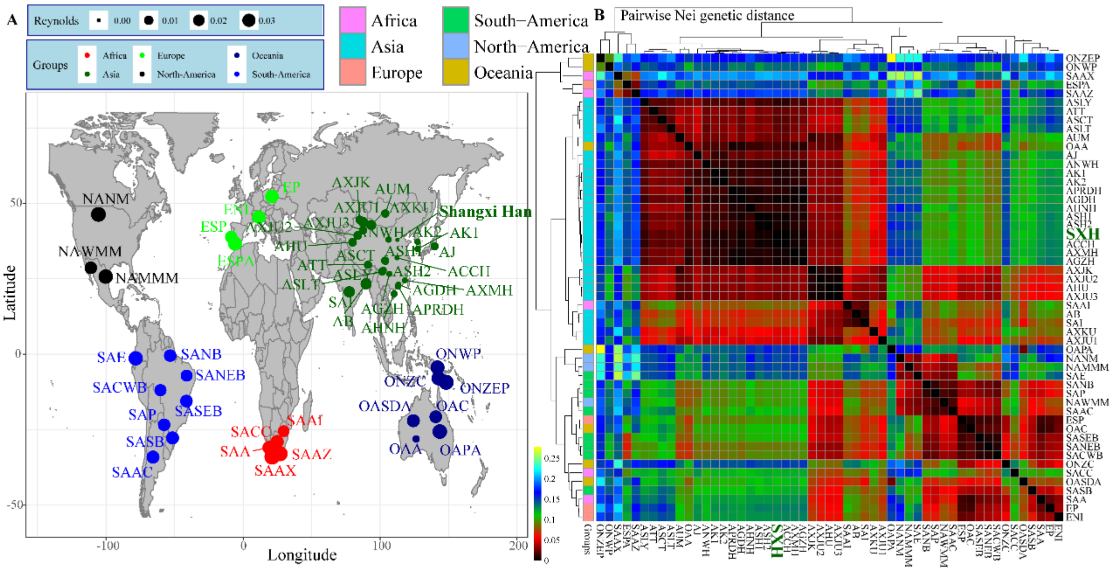

Figure S2. Sites and pairwise Reynolds genetic distance of newly studied Shanxi Han and 52 worldwide reference populations (**A**). Populations from different continents are designed by colored circles. Pairwise Nei genetic distance distribution among 53 worldwide populations (**B**).

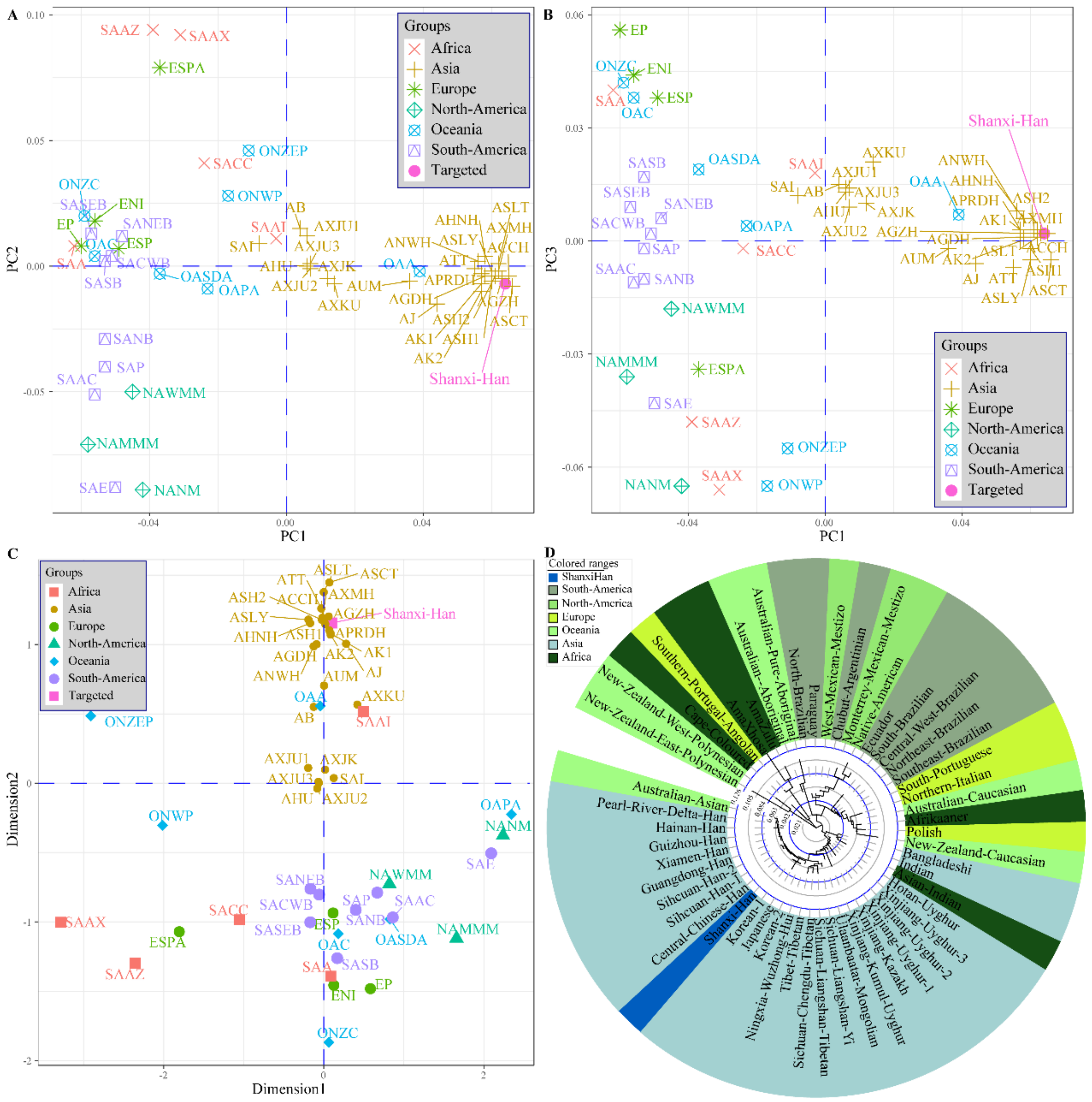

Figure S3. Population genetic analyses and phylogenetic relationship reconstruction among 53 worldwide populations. Principal component analysis with Shanxi Han projected onto worldwide reference populations based on the first two components (A) and the combination of the first and third components (B). Multidimensional scaling plot result displays the genetic similarities and differences (C). Phylogenetic relationship revealed by the neighbor-joining tree (D).

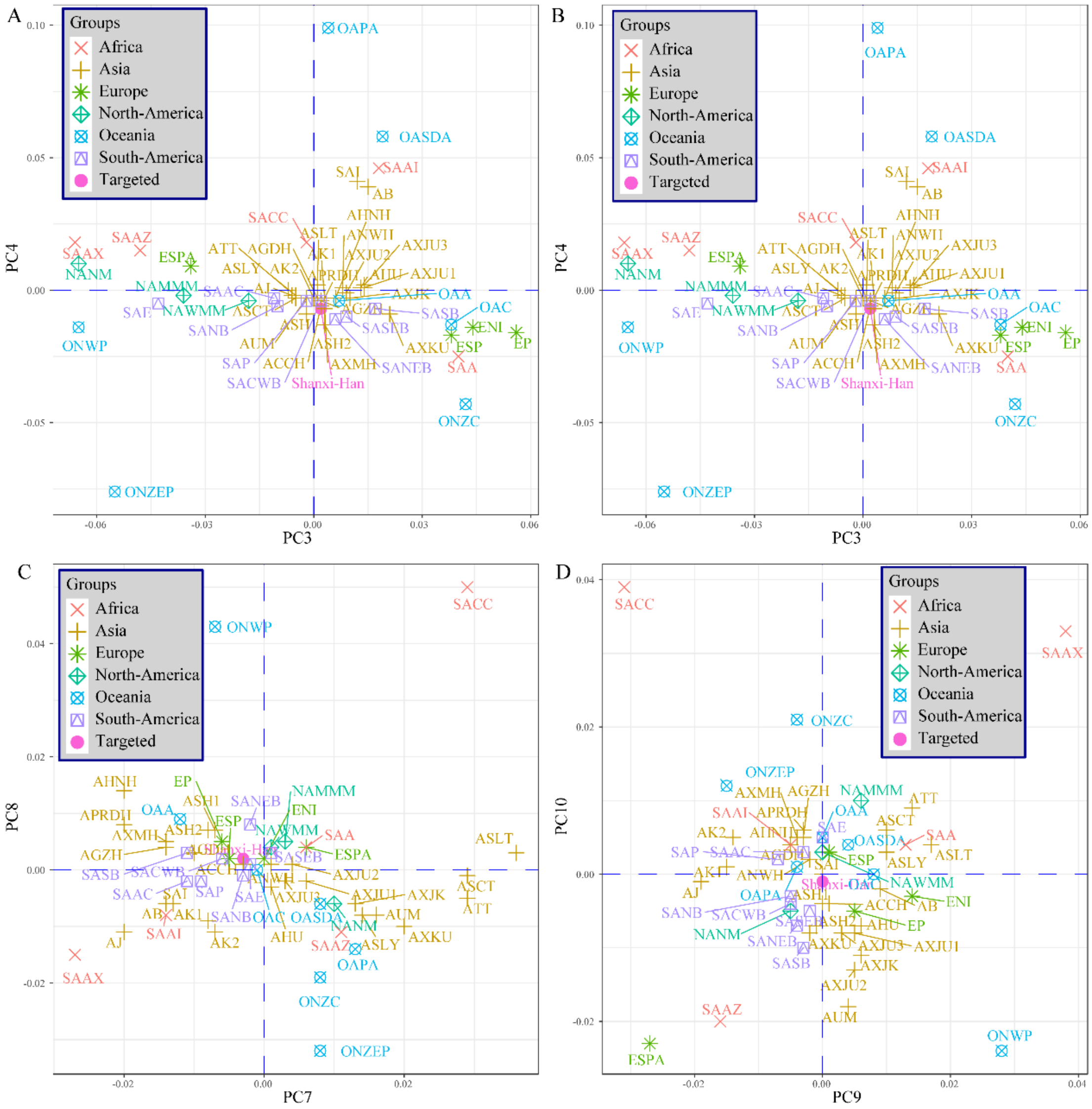

Figure S4. The third to the tenth components in the principal component analysis revealed the genetic similarities and differences among 53 worldwide populations.

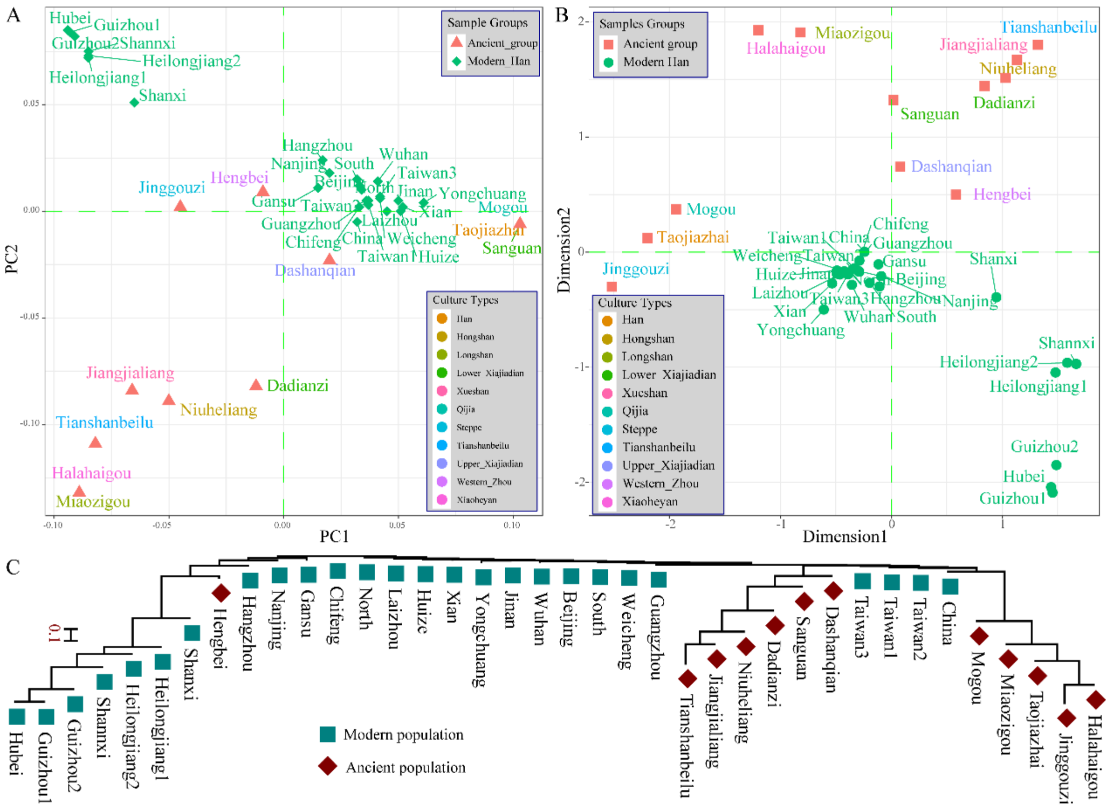

Figure S5. Phylogenetic relationship between Neolithic ancient and modern populations on the basis of the Y-chromosome variations. (A). The first two components revealed the genetic affinity between 12 ancient and 26 modern Chinese populations. (B). Two-dimensional scaling plots among 38 populations. (C). Phylogenetic tree among ancient and modern populations reconstructed based on the linearized Fst genetic distance.

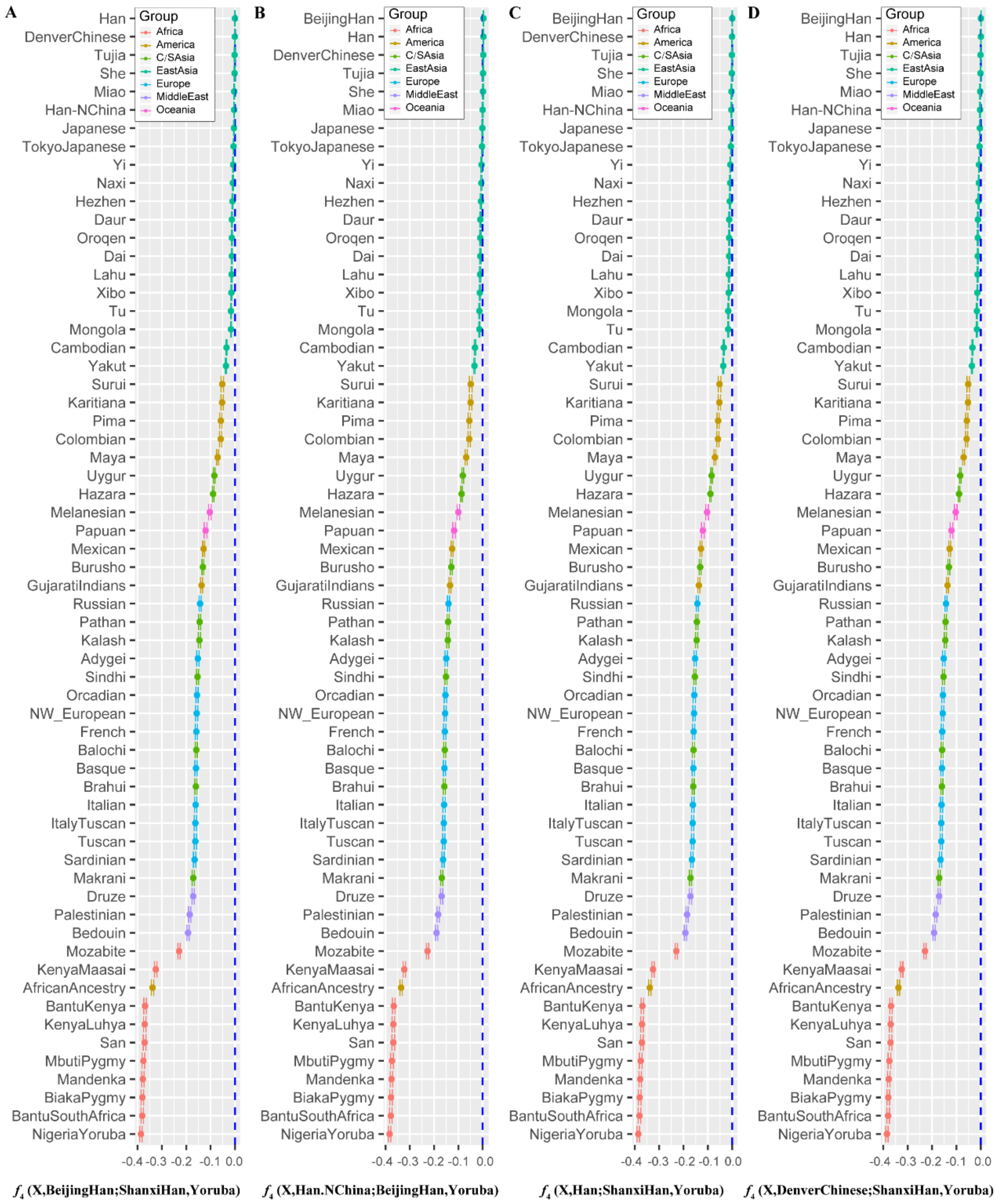

Figure S6. Shared genetic components with Shanxi Han between other Han Chinese groups (A, Beijing Han; B, NChia; C, Han; D, Denver Chinese) and worldwide reference populations.

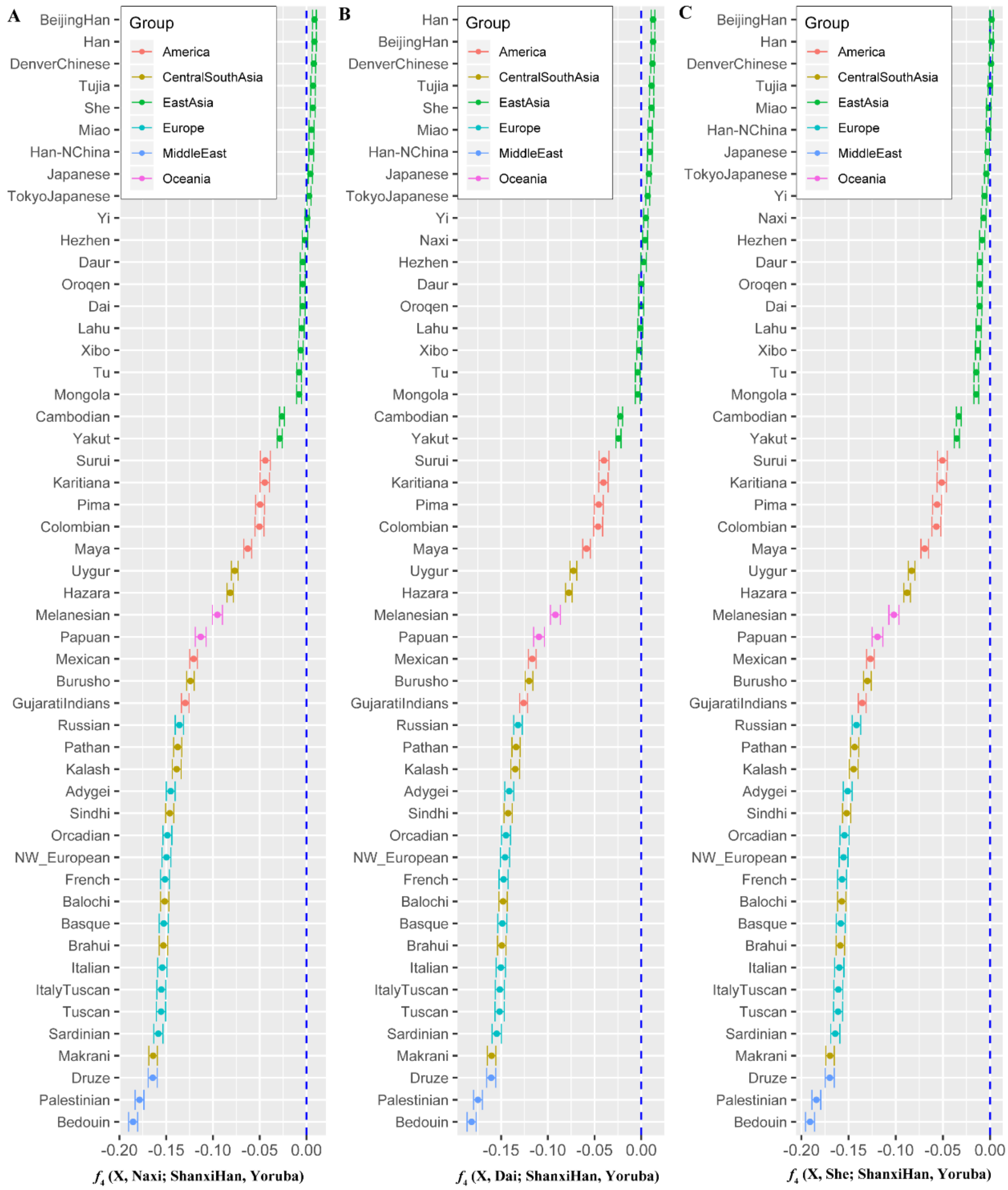

Figure S7. Shared genetic components with Shanxi Han between Tibeto-Burman-speaking Naxi (A), Tai-Kadai-speaking Dai (B), Hmong-Mien-speaking She (C) and worldwide reference populations.

Figure S7A

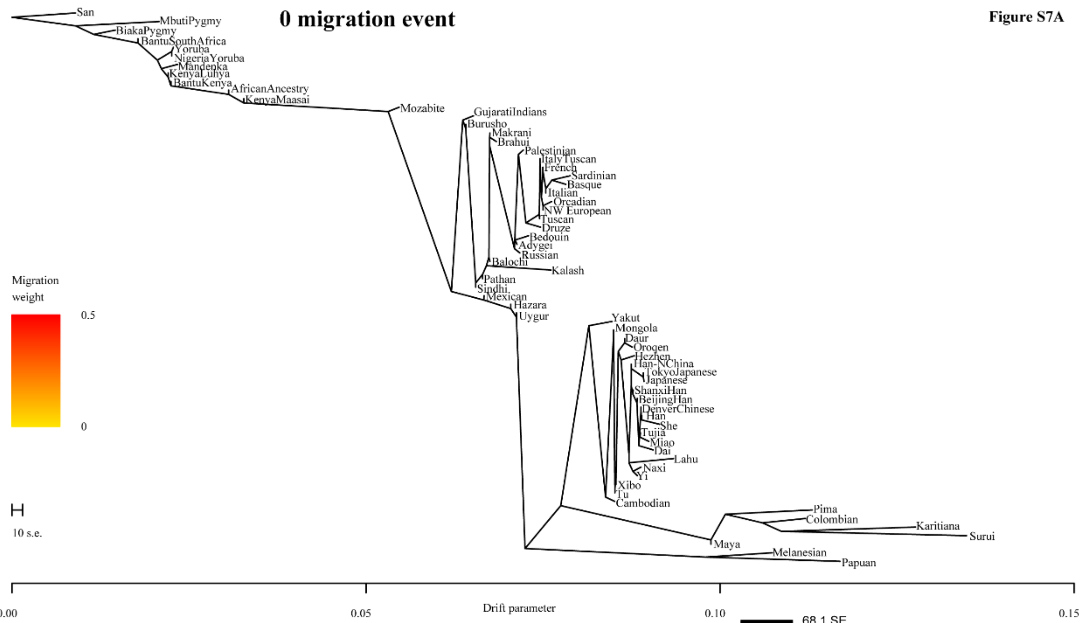

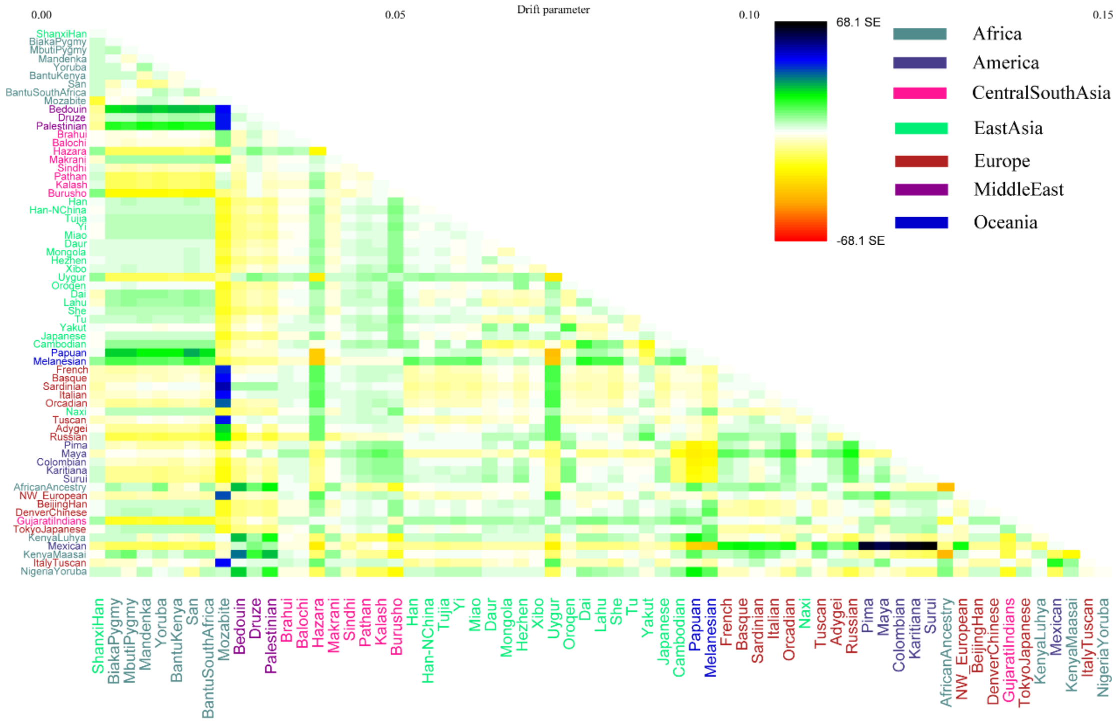

Figure S8A

Figure S7B

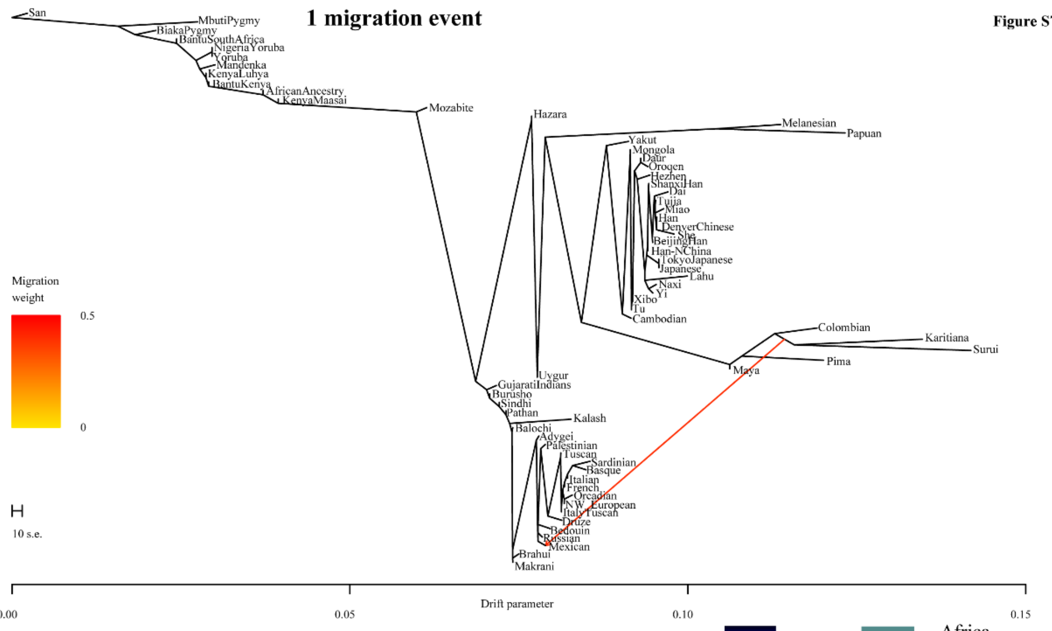

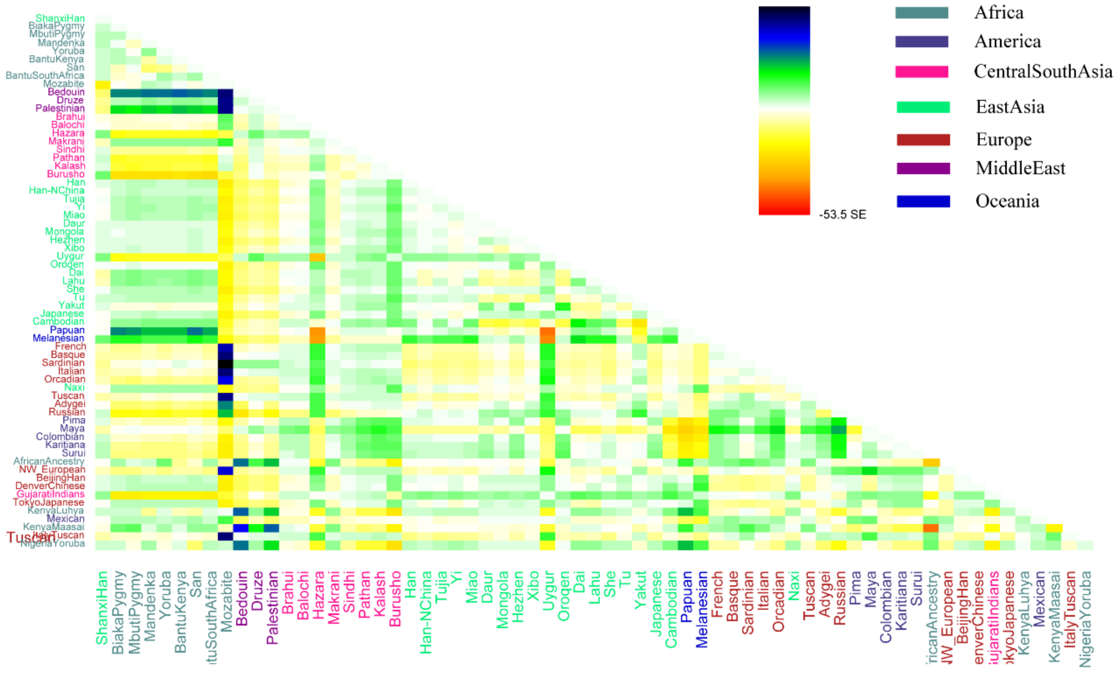

Figure S8B

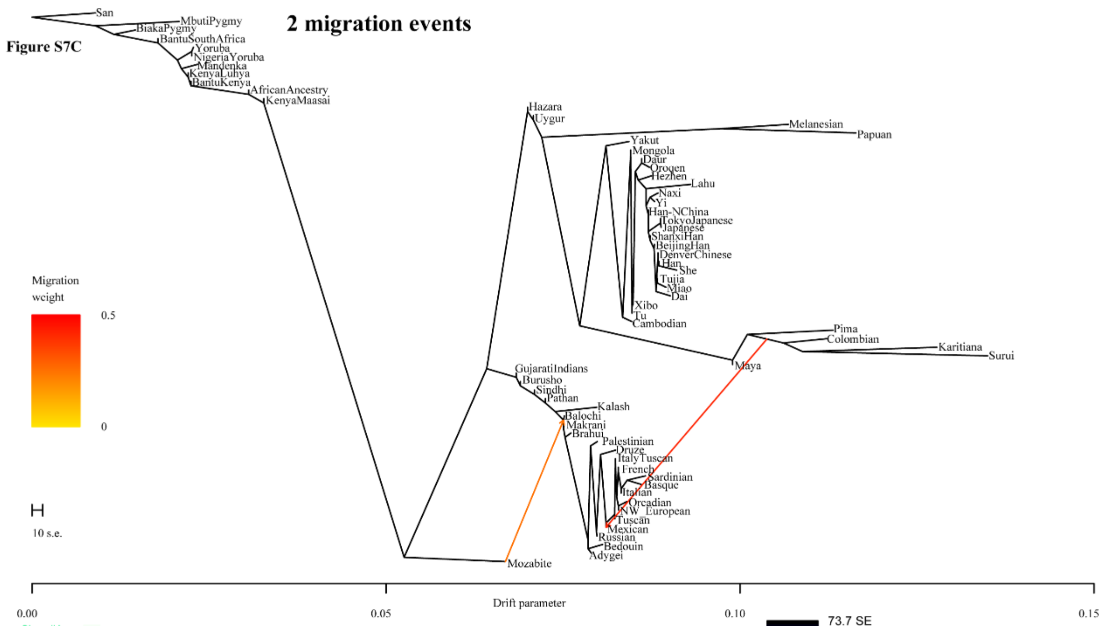

Figure S7C

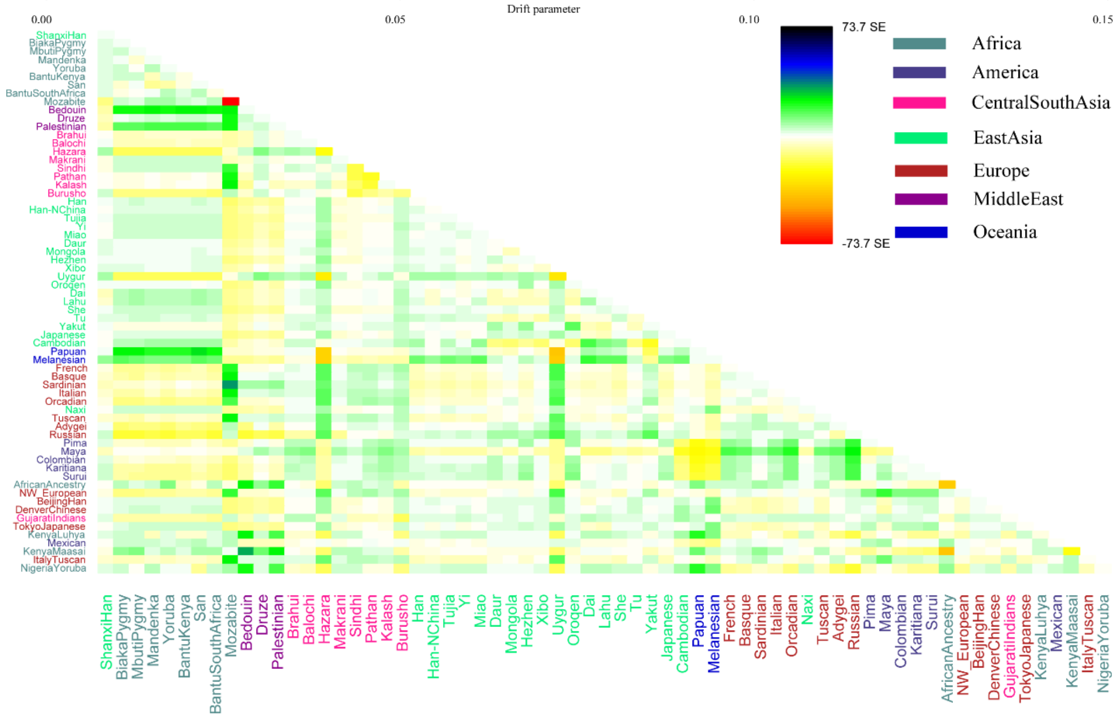

Figure S8C

Figure S7D

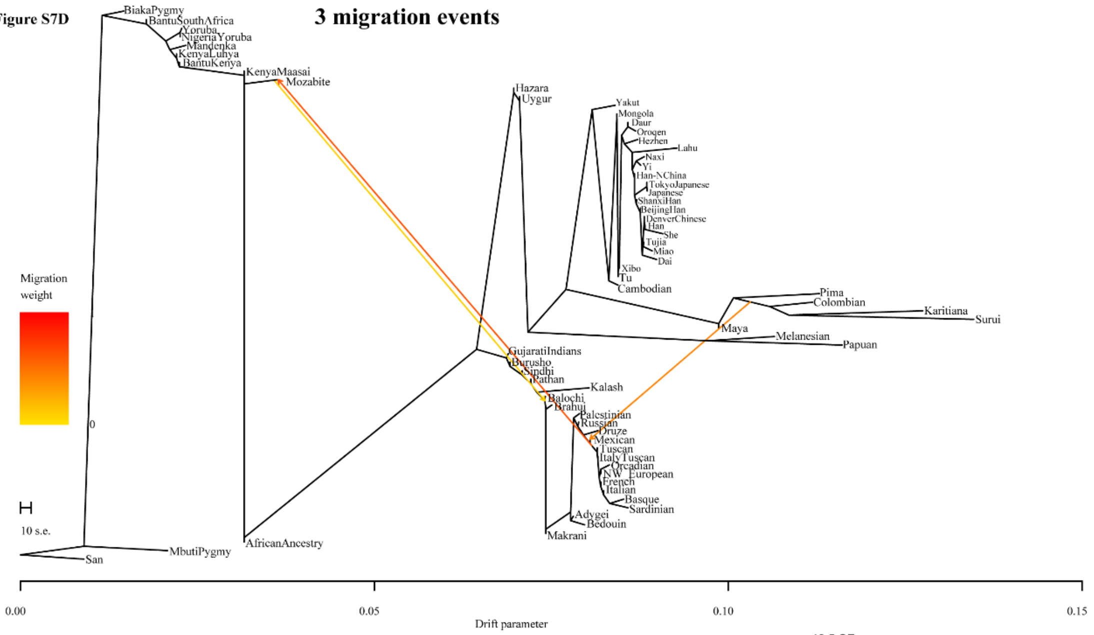

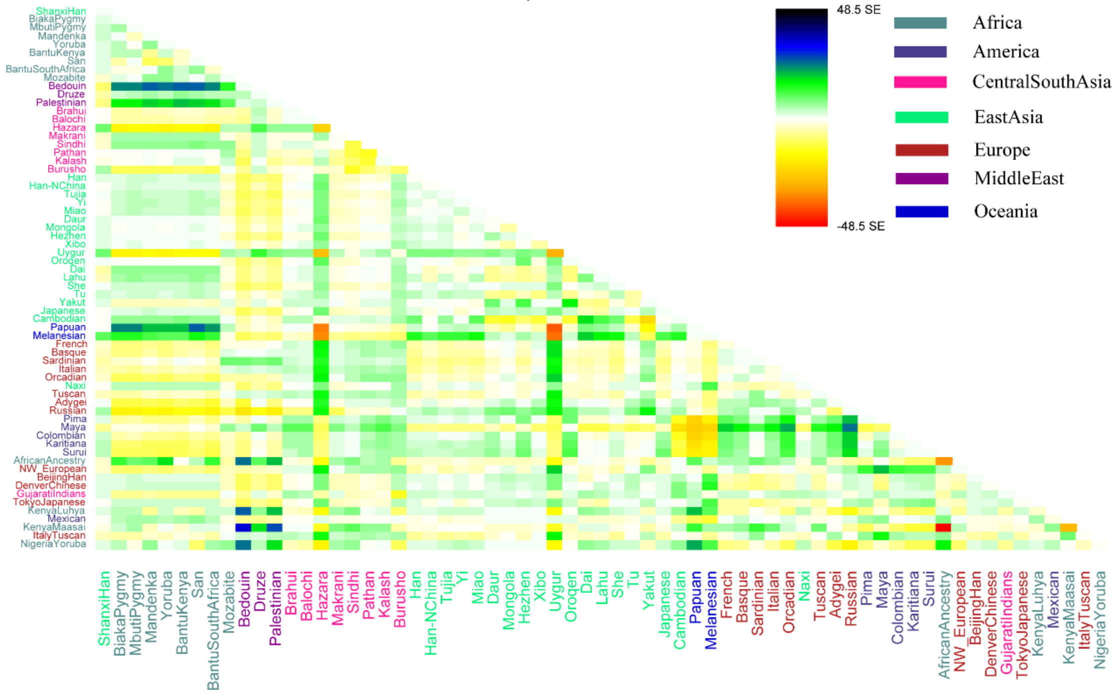

Figure S8D

Figure S7E

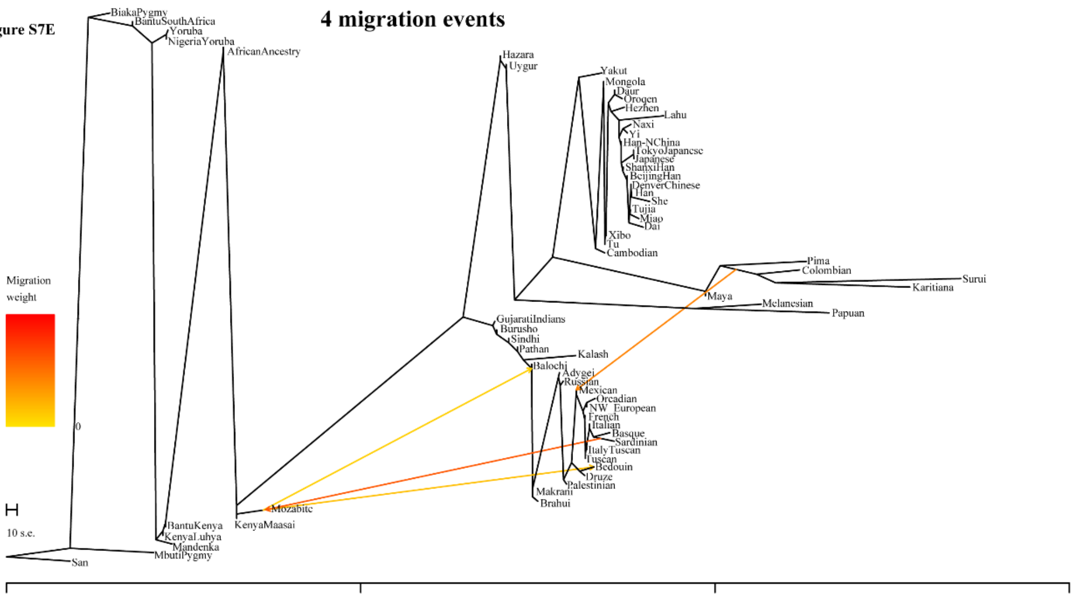

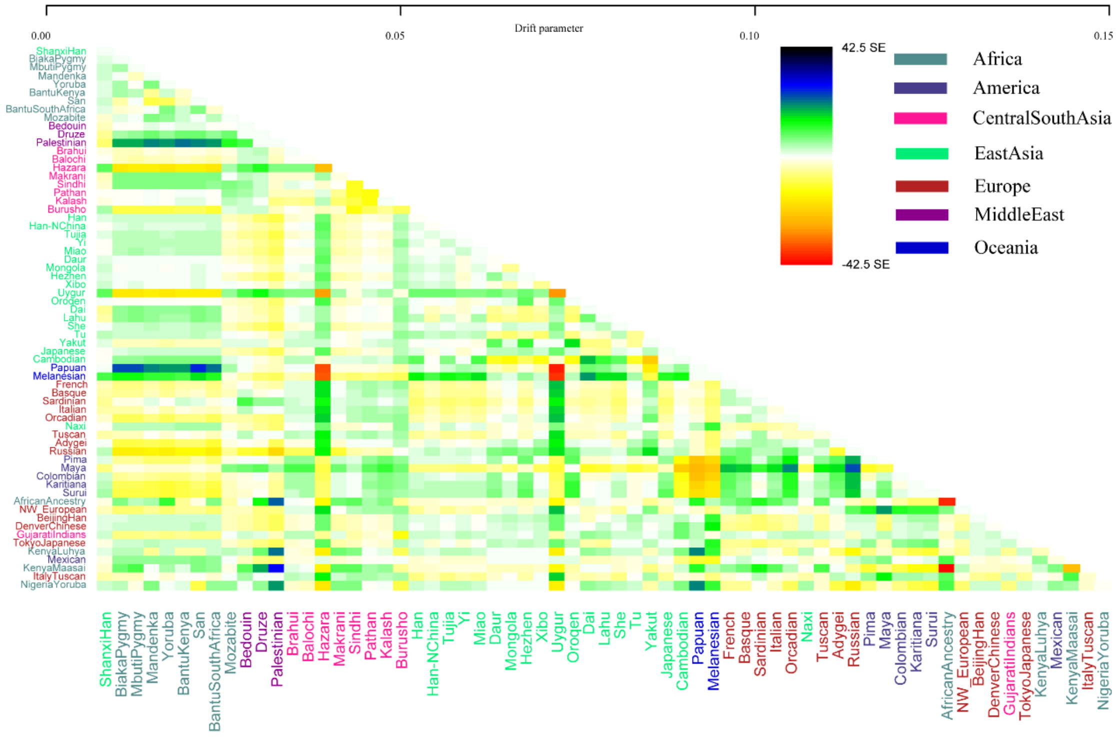

Figure S8E

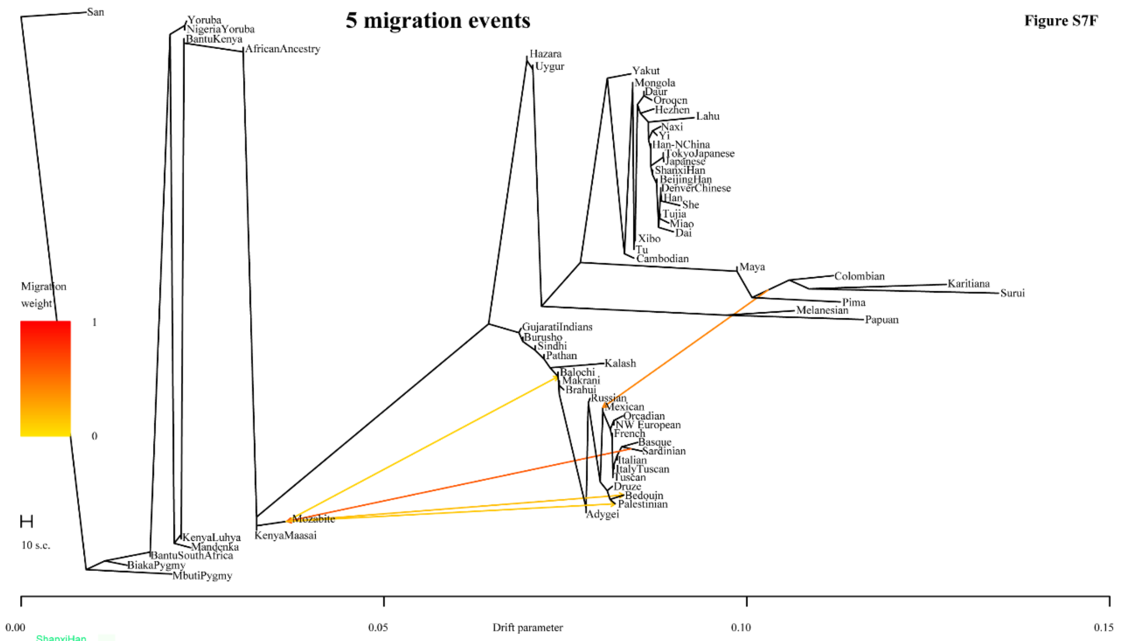
5 migration events
Figure S7F
Migration weight
1
0
10 s.e.
San
Yoruba
NigeriaYoruba
BantuKenya
AfricanAncestry
KenyaLuhya
Mandenka
BantuSouthAfrica
BiakaPygmy
MbutiPygmy
Mozabite
KenyaMaasai
Hazara
Uygur
Yakut
Mongola
Daur
Oroqen
Hezhen
Lahu
Naxi
Yi
Han-NChina
TokyoJapanese
Japanese
ShanxiHan
Han
BeijingHan
DenverChinese
She
Tujia
Miao
Dai
Xibo
Tu
Cambodian
Maya
Colombian
Karitiana
Surui
Pima
Melanesian
Papuan
GujaratiIndians
Burusho
Sindhi
Pathan
Kalash
Balochi
Makrani
Brahui
Russian
Mexican
Orcadian
NW European
French
Basque
Sardinian
Italian
ItalyTuscan
Tuscan
Druze
Bedouin
Palestinian
Adygei
0.00
0.05
Drift parameter
0.10
0.15

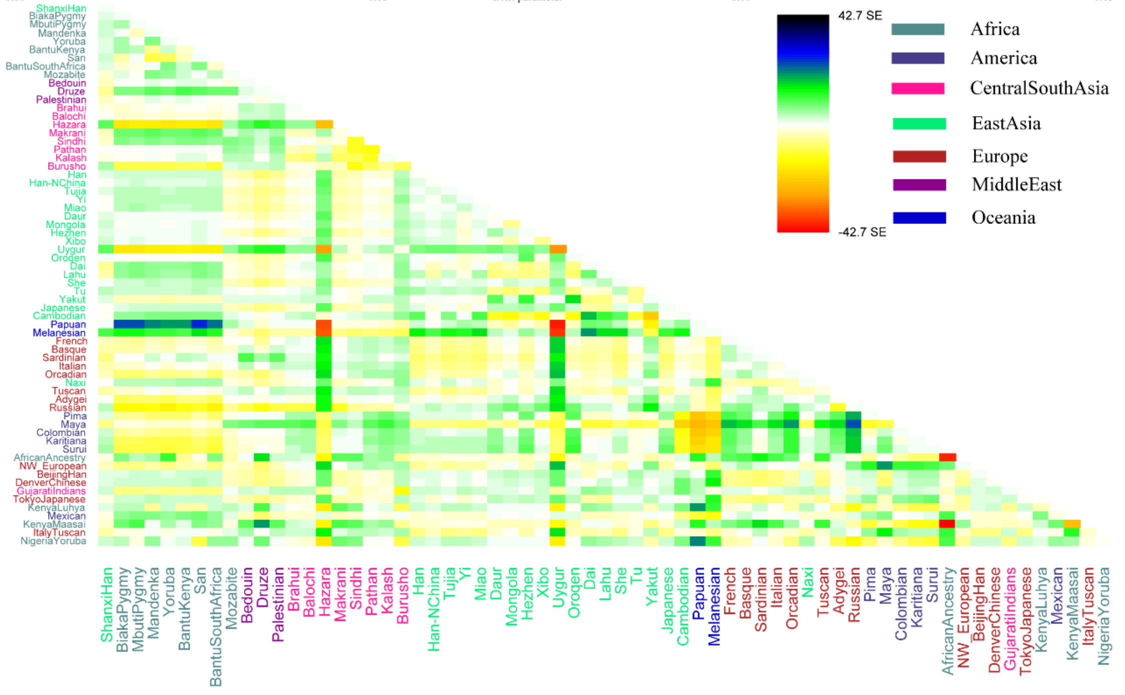
42.7 SE
-42.7 SE
Africa
America
CentralSouthAsia
EastAsia
Europe
MiddleEast
Oceania
ShanxiHan
BiakaPygmy
MbutiPygmy
Mandenka
Yoruba
BantuKenya
San
BantuSouthAfrica
Mozabite
Bedouin
Druze
Palestinian
Brahui
Balochi
Hazara
Makrani
Sindhi
Pathan
Kalash
Burusho
Han
Han-NChina
Tujia
Yi
Miao
Daur
Mongola
Hezhen
Xibo
Uygur
Oroqen
Dai
Lahu
She
Tu
Yakut
Japanese
Cambodian
Papuan
Melanesian
French
Basque
Sardinian
Italian
Orcadian
Naxi
Tuscan
Adygei
Russian
Pima
Maya
Colombian
Karitiana
Surui
AfricanAncestry
NW_European
BeijingHan
DenverChinese
GujaratiIndians
TokyoJapanese
KenyaLuhya
Mexican
KenyaMaasai
ItalyTuscan
NigeriaYoruba

Figure S8F

Figure S7G

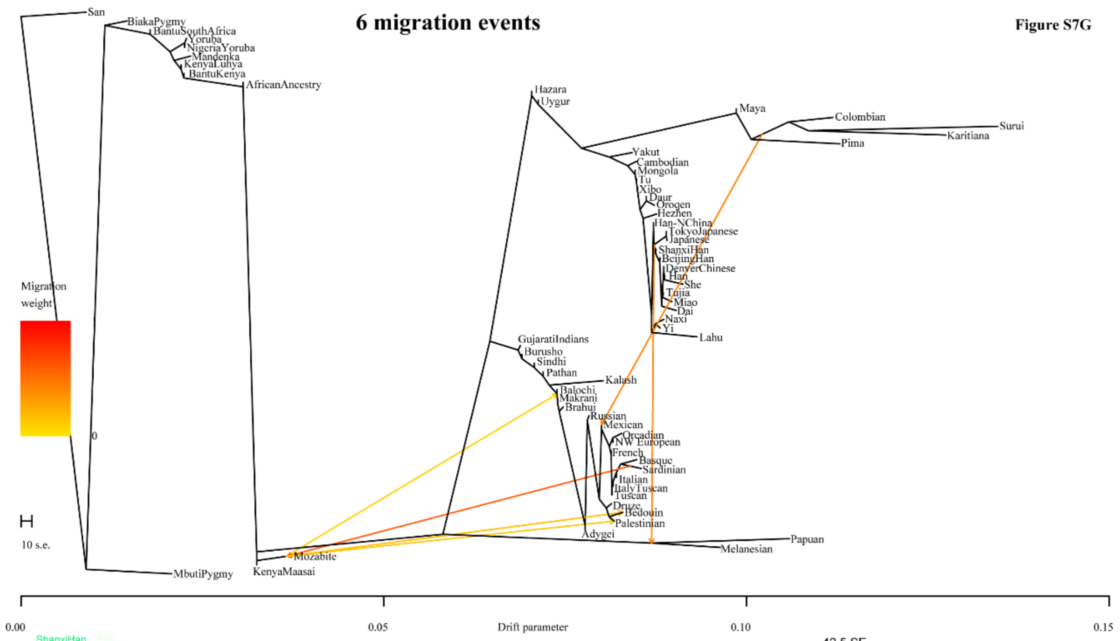

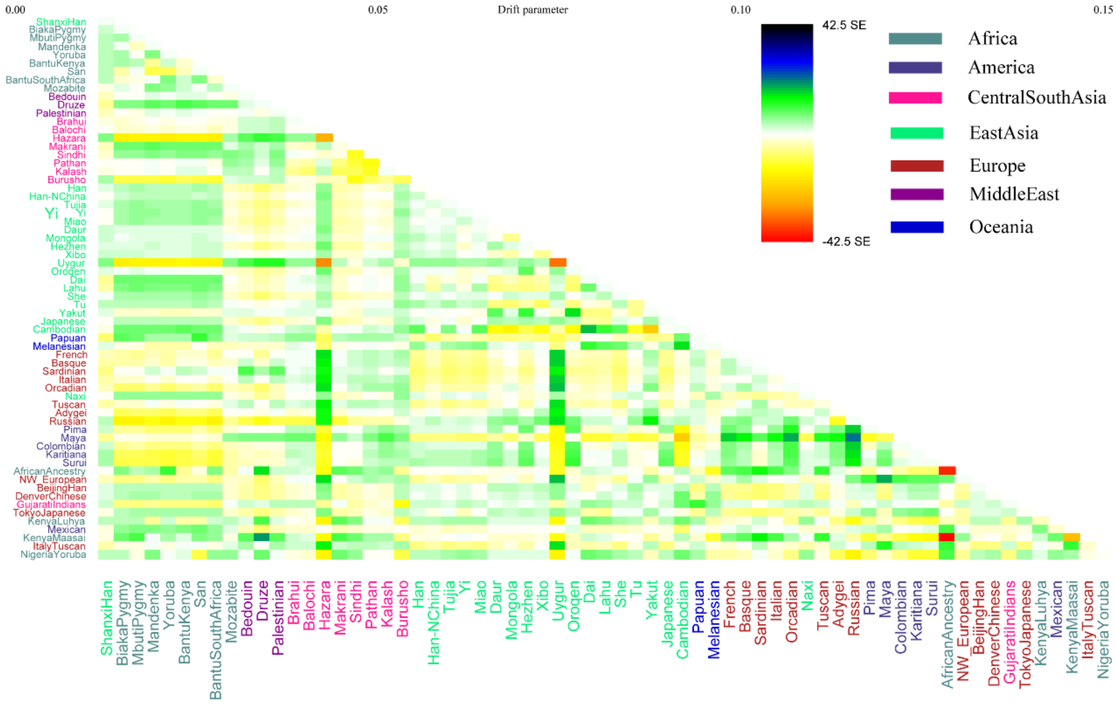

Figure S8G

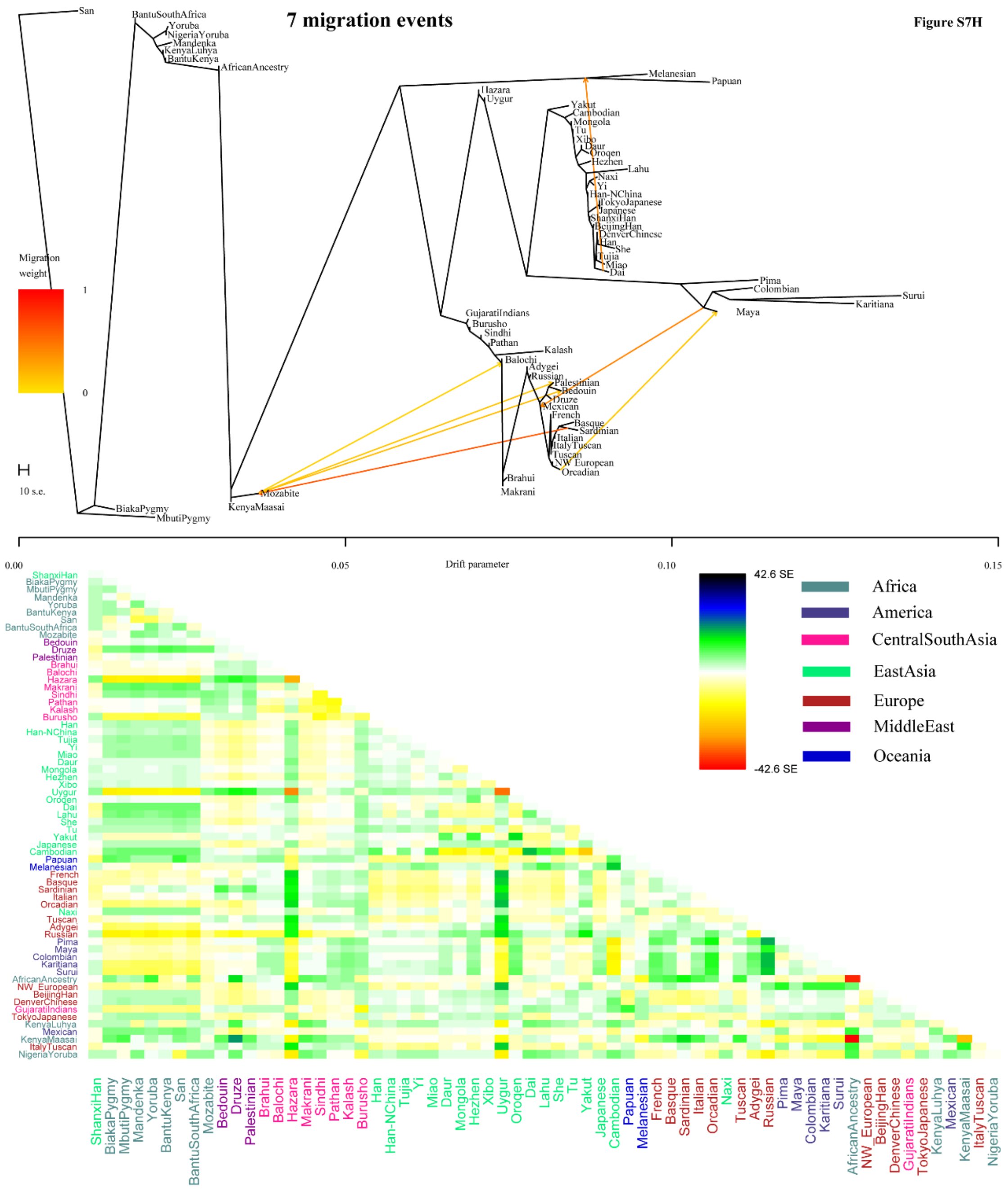

7 migration events
Figure S7H
Migration weight
Drift parameter
10 s.e.
42.6 SE
-42.6 SE
Africa
America
CentralSouthAsia
EastAsia
Europe
MiddleEast
Oceania

Figure S8H

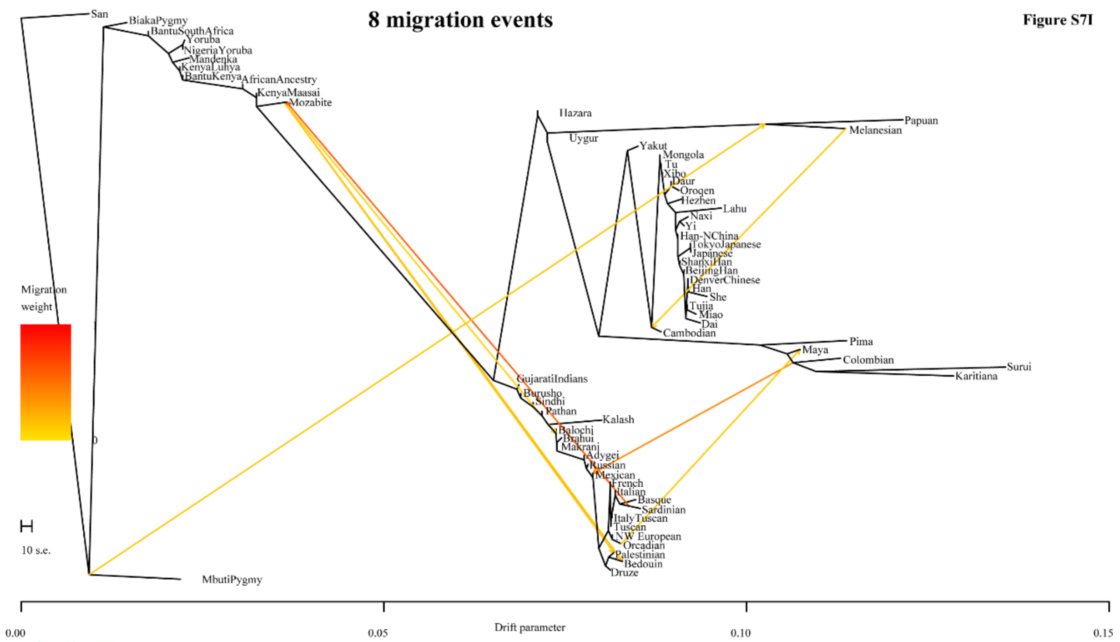
8 migration events
Figure S7I
Migration weight
10 s.e.
Drift parameter
0.00
0.05
0.10
0.15

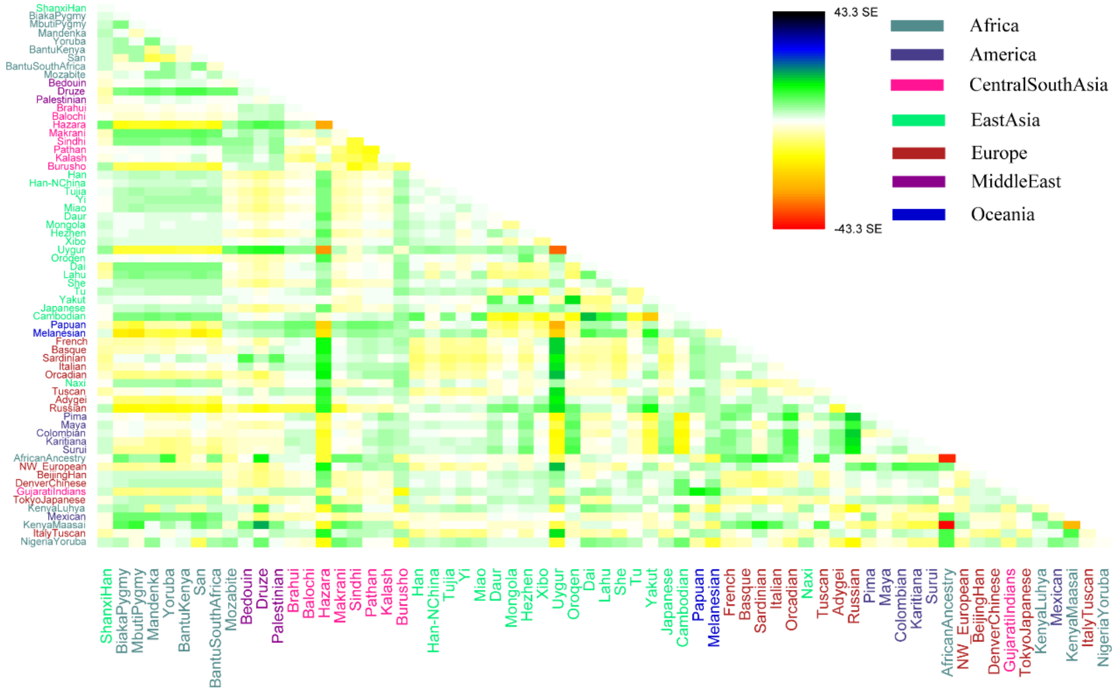
43.3 SE
-43.3 SE
Africa
America
CentralSouthAsia
EastAsia
Europe
MiddleEast
Oceania

Figure S8I

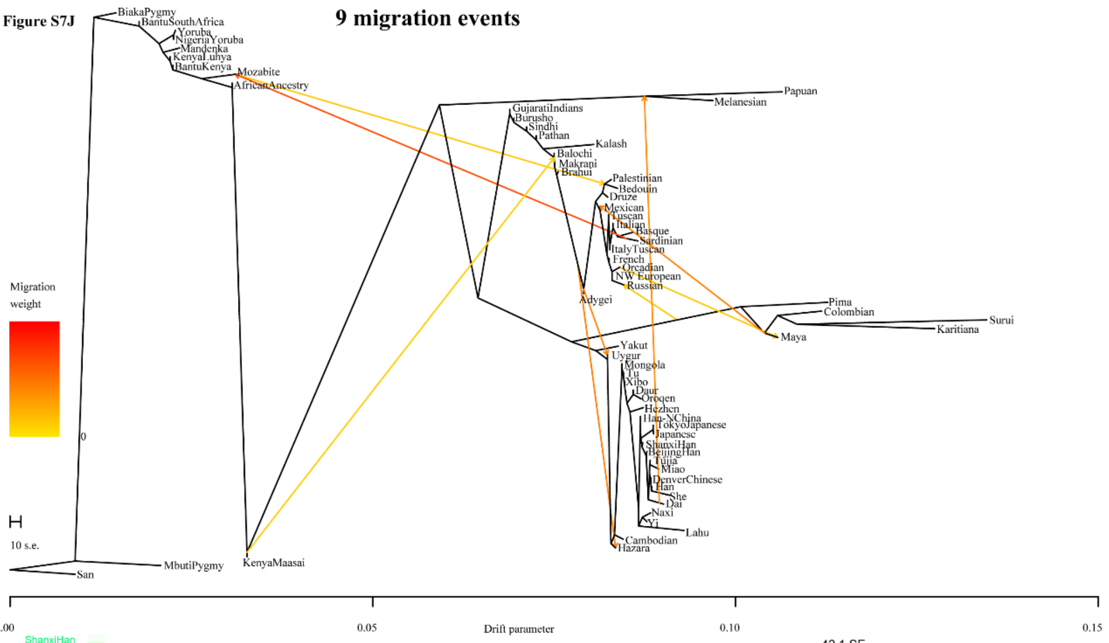

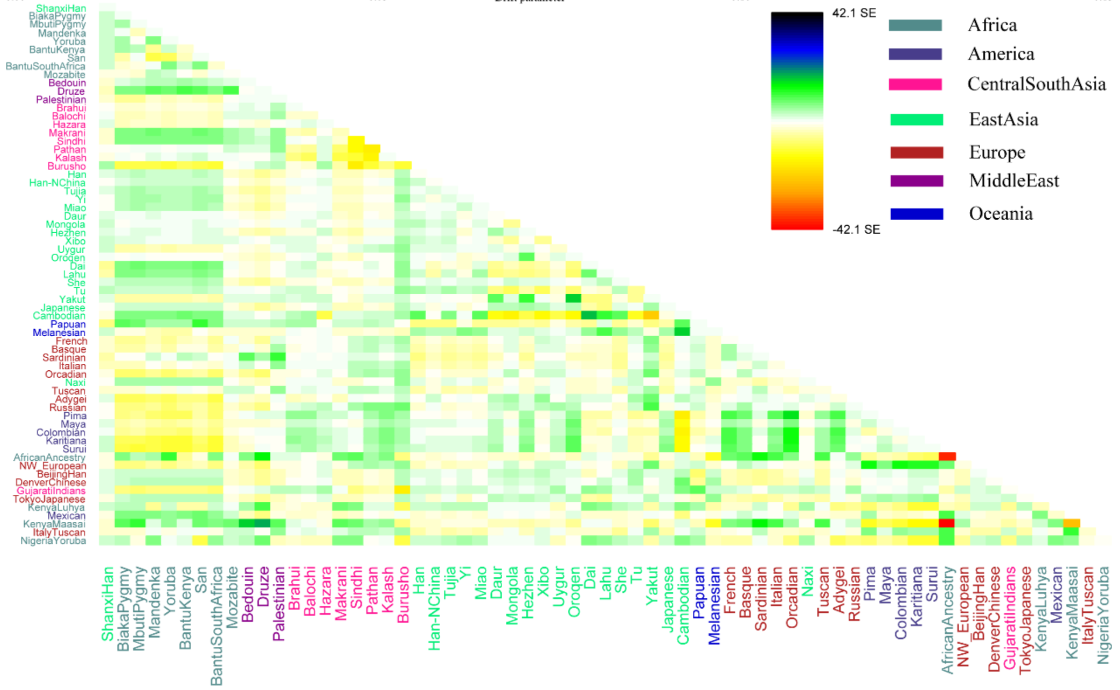

Figure S8J

Figure S8. Inference of separation of worldwide populations via the phylogenetic relationship and population splits with the predefined 0~9 migration events.

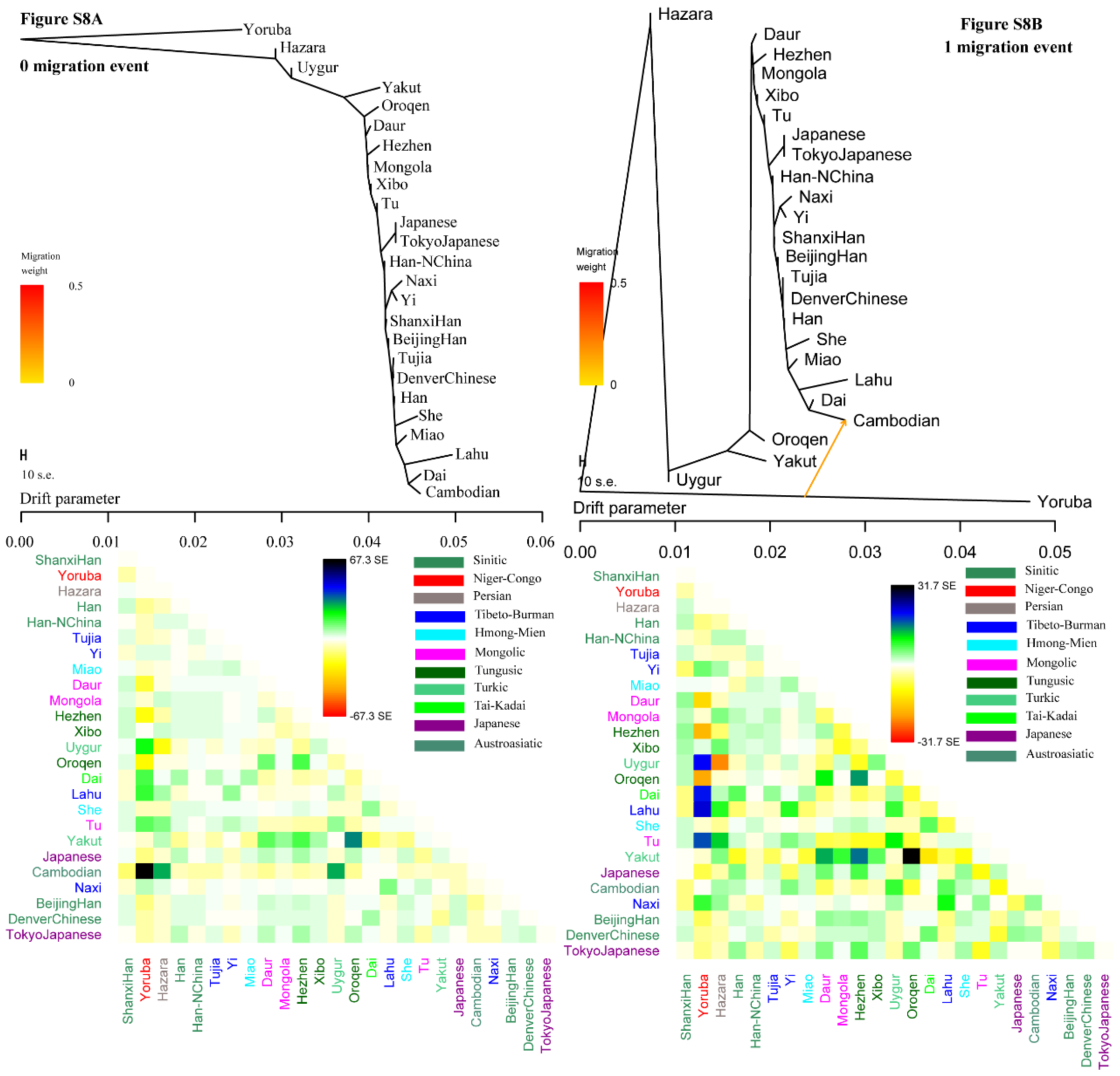
Figure S8A
0 migration event
Yoruba
Hazara
Uygur
Yakut
Oroqen
Daur
Hezhen
Mongola
Xibo
Tu
Japanese
TokyoJapanese
Han-NChina
Naxi
Yi
ShanxiHan
BeijingHan
Tujia
DenverChinese
Han
She
Miao
Lahu
Dai
Cambodian
Migration weight
0.5
0
10 s.e.
Drift parameter
0.00 0.01 0.02 0.03 0.04 0.05 0.06
Figure S8B
1 migration event
Hazara
Daur
Hezhen
Mongola
Xibo
Tu
Japanese
TokyoJapanese
Han-NChina
Naxi
Yi
ShanxiHan
BeijingHan
Tujia
DenverChinese
Han
She
Miao
Lahu
Dai
Cambodian
Oroqen
Yakut
Uygur
Yoruba
Migration weight
0.5
0
10 s.e.
Drift parameter
0.00 0.01 0.02 0.03 0.04 0.05
67.3 SE
-67.3 SE
31.7 SE
-31.7 SE
Sinitic
Niger-Congo
Persian
Tibeto-Burman
Hmong-Mien
Mongolic
Tungusic
Turkic
Tai-Kadai
Japanese
Austroasiatic
ShanxiHan
Yoruba
Hazara
Han
Han-NChina
Tujia
Yi
Miao
Daur
Mongola
Hezhen
Xibo
Uygur
Oroqen
Dai
Lahu
She
Tu
Yakut
Japanese
Cambodian
Naxi
BeijingHan
DenverChinese
TokyoJapanese

Figure S9AB

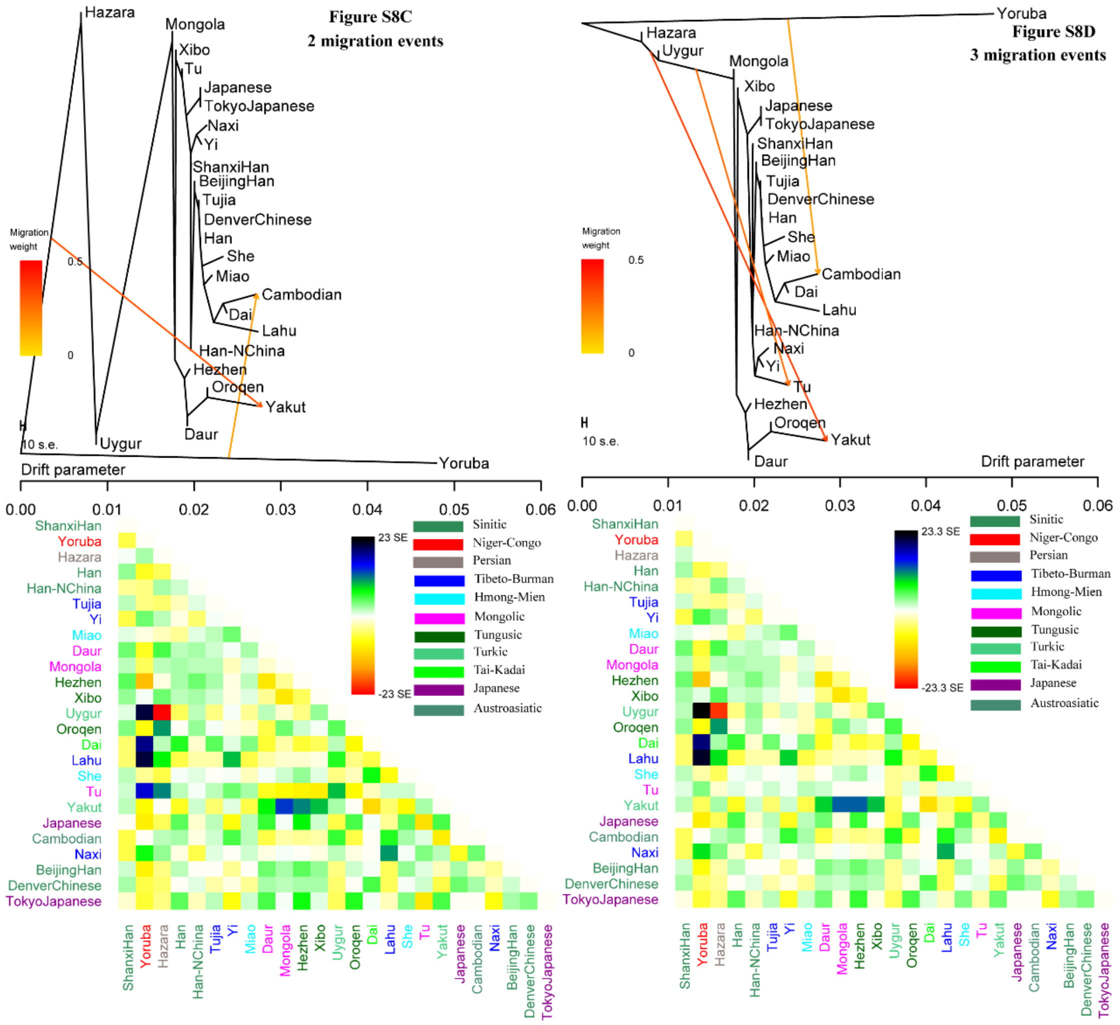

Figure S8C
2 migration events
Figure S8D
3 migration events
Migration weight
Drift parameter
10 s.e.
Hazara
Mongola
Xibo
Tu
Japanese
TokyoJapanese
Naxi
Yi
ShanxiHan
BeijingHan
Tujia
DenverChinese
Han
She
Miao
Cambodian
Dai
Lahu
Han-NChina
Hezhen
Oroqen
Yakut
Daur
Uygur
Yoruba
23 SE
-23 SE
23.3 SE
-23.3 SE
Sinitic
Niger-Congo
Persian
Tibeto-Burman
Hmong-Mien
Mongolic
Tungusic
Turkic
Tai-Kadai
Japanese
Austroasiatic

Figure S9CD

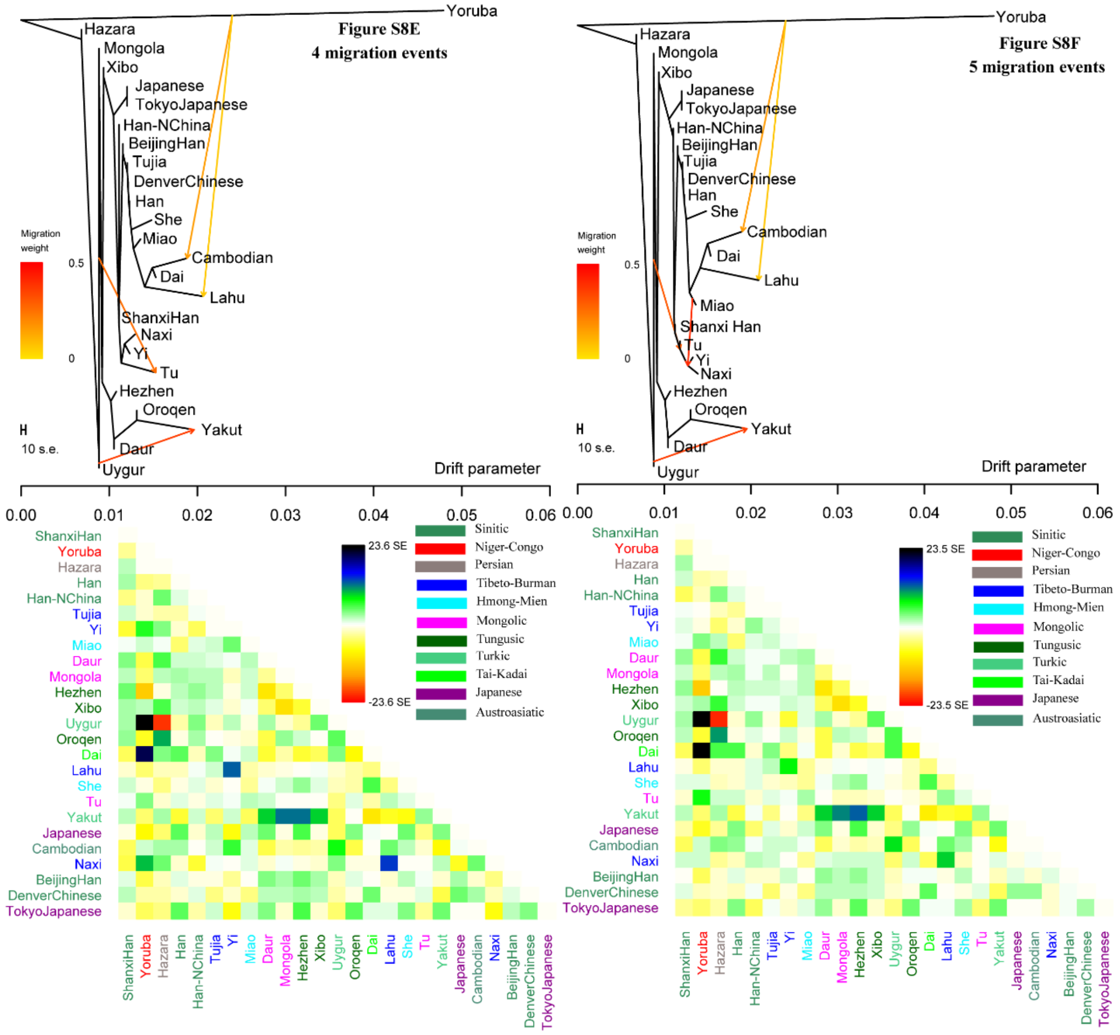
Figure S8E
4 migration events
Figure S8F
5 migration events
Yoruba
Hazara
Mongola
Xibo
Japanese
TokyoJapanese
Han-NChina
BeijingHan
Tujia
DenverChinese
Han
She
Miao
Cambodian
Dai
Lahu
ShanxiHan
Shanxi Han
Naxi
Yi
Tu
Hezhen
Oroqen
Yakut
Daur
Uygur
Migration weight
0.5
0
10 s.e.
Drift parameter
0.00
0.01
0.02
0.03
0.04
0.05
0.06
23.6 SE
-23.6 SE
23.5 SE
-23.5 SE
Sinitic
Niger-Congo
Persian
Tibeto-Burman
Hmong-Mien
Mongolic
Tungusic
Turkic
Tai-Kadai
Japanese
Austroasiatic

Figure S9EF

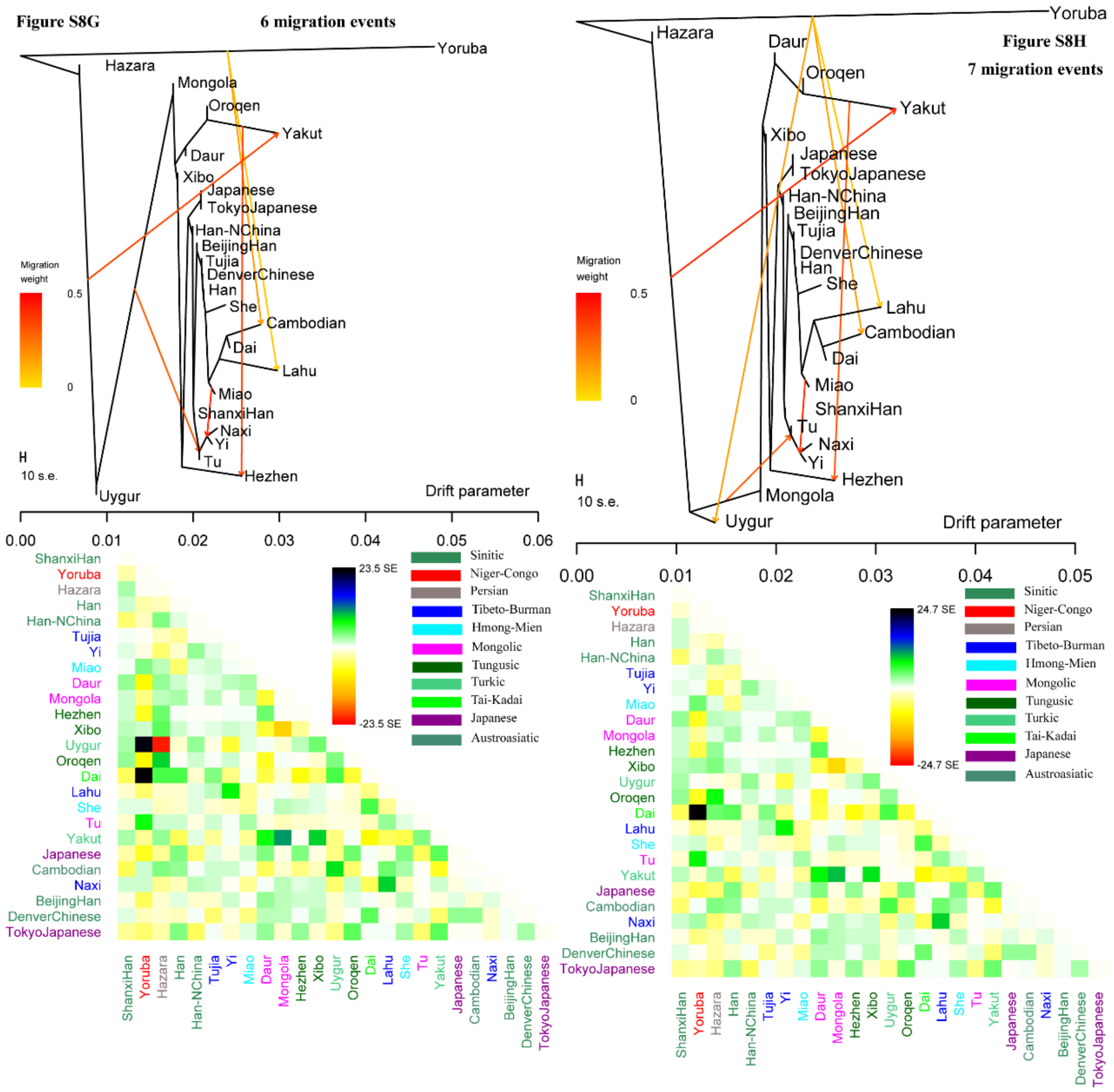
Figure S8G
6 migration events
Yoruba
Hazara
Mongola
Oroqen
Yakut
Daur
Xibo
Japanese
TokyoJapanese
Han-NChina
BeijingHan
Tujia
DenverChinese
Han
She
Cambodian
Dai
Lahu
Miao
ShanxiHan
Naxi
Yi
Tu
Hezhen
Uygur
Migration weight
0.5
0
10 s.e.
Drift parameter
0.00
0.01
0.02
0.03
0.04
0.05
0.06
23.5 SE
-23.5 SE
Sinitic
Niger-Congo
Persian
Tibeto-Burman
Hmong-Mien
Mongolic
Tungusic
Turkic
Tai-Kadai
Japanese
Austroasiatic
Figure S8H
7 migration events
Mongola
24.7 SE
-24.7 SE

Figure S9GH

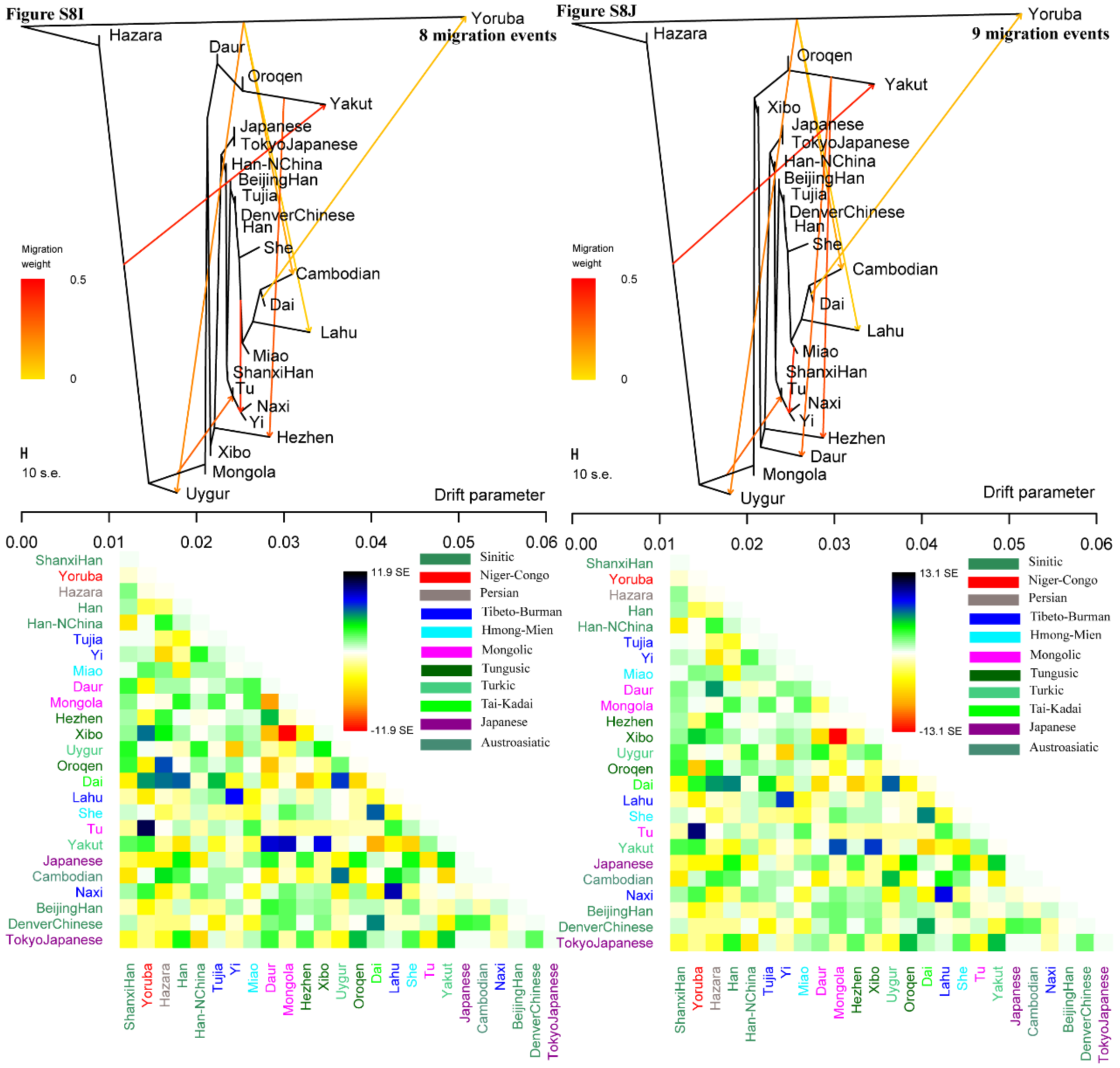
Figure S8I
8 migration events
Yoruba
Hazara
Daur
Oroqen
Yakut
Japanese
TokyoJapanese
Han-NChina
BeijingHan
Tujia
DenverChinese
Han
She
Cambodian
Dai
Lahu
Miao
ShanxiHan
Tu
Naxi
Yi
Hezhen
Xibo
Mongola
Uygur
Migration weight
0.5
0
10 s.e.
Drift parameter
0.00 0.01 0.02 0.03 0.04 0.05 0.06
11.9 SE
-11.9 SE
Figure S8J
9 migration events
13.1 SE
-13.1 SE
Sinitic
Niger-Congo
Persian
Tibeto-Burman
Hmong-Mien
Mongolic
Tungusic
Turkic
Tai-Kadai
Japanese
Austroasiatic

Figure S9IJ

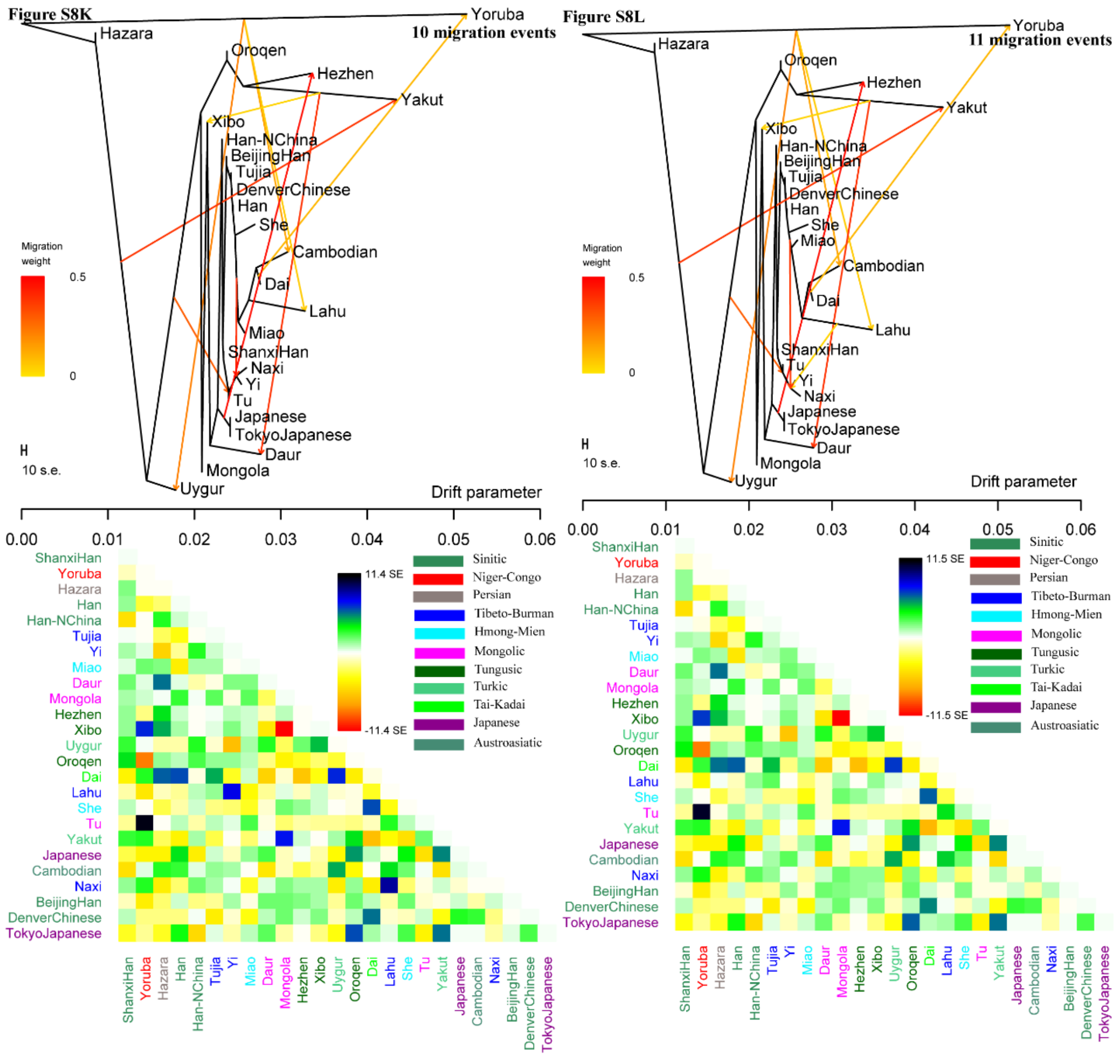

Figure S8K
10 migration events
Yoruba
Hazara
Oroqen
Hezhen
Yakut
Xibo
Han-NChina
BeijingHan
Tujia
DenverChinese
Han
She
Cambodian
Dai
Lahu
Miao
ShanxiHan
Naxi
Yi
Tu
Japanese
TokyoJapanese
Daur
Mongola
Uygur
Migration weight
0.5
0
10 s.e.
Drift parameter
0.00
0.01
0.02
0.03
0.04
0.05
0.06
11.4 SE
-11.4 SE
Sinitic
Niger-Congo
Persian
Tibeto-Burman
Hmong-Mien
Mongolic
Tungusic
Turkic
Tai-Kadai
Japanese
Austroasiatic
Figure S8L
11 migration events
11.5 SE
-11.5 SE

Figure S9KL

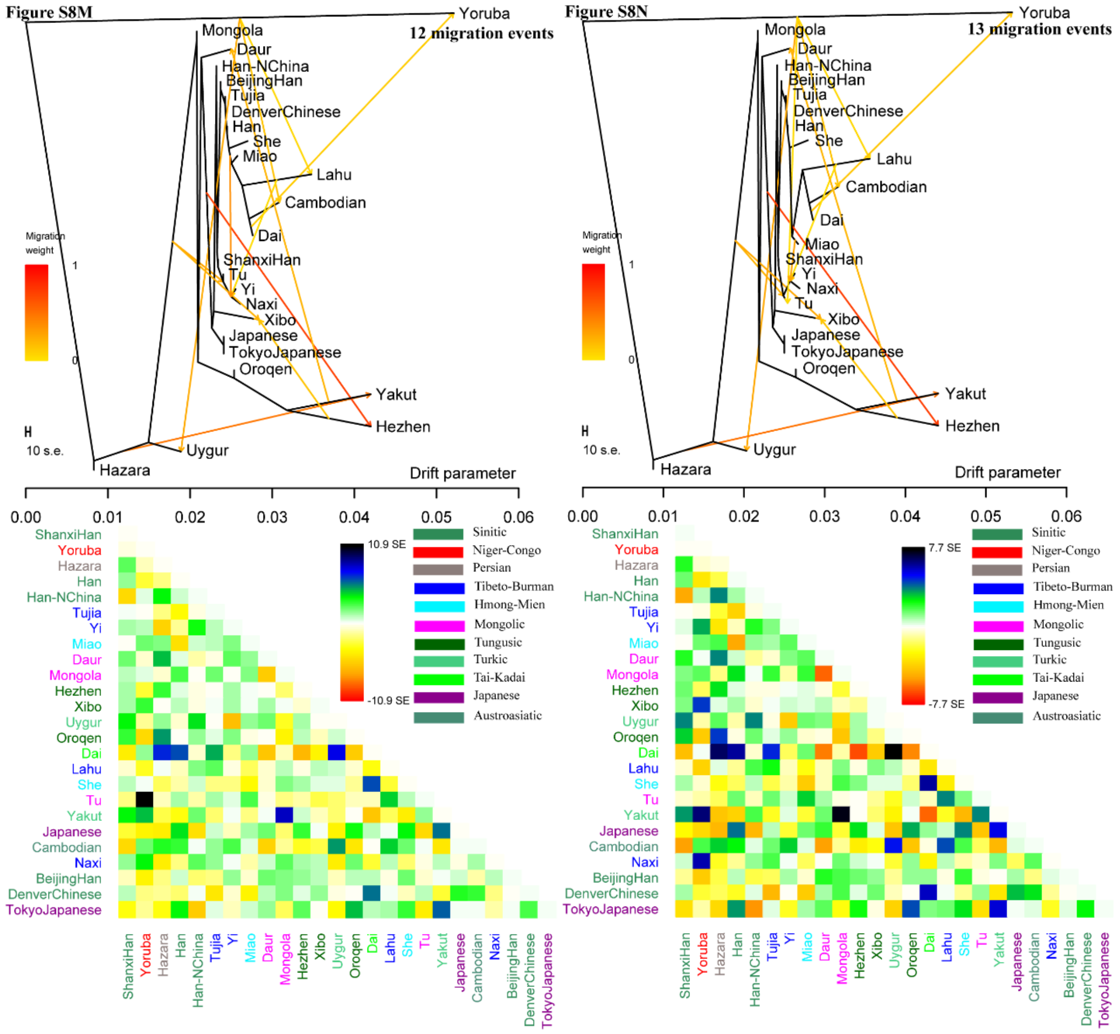

Figure S9MN

Figure S9. Inference of separation of Asian populations via the phylogenetic relationship and population splits with the predefined 0~13 migration events.

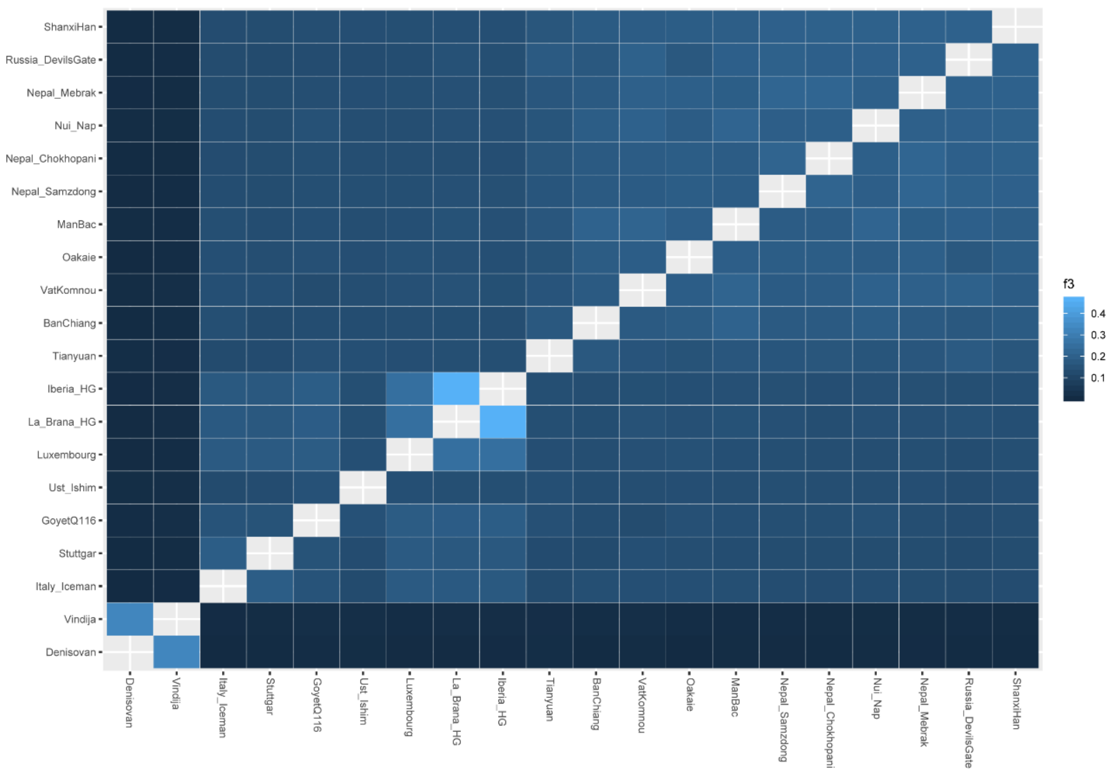

Figure S10. Shared genetic drift among Shanxi Han and other 19 worldwide ancient populations.
